# Biofilm formation on the surface of monazite and xenotime during bioleaching

**DOI:** 10.1111/1751-7915.14260

**Published:** 2023-06-08

**Authors:** Arya van Alin, Melissa K. Corbett, Homayoun Fathollahzadeh, M. Christian Tjiam, William D. A. Rickard, Xiao Sun, Andrew Putnis, Jacques Eksteen, Anna H. Kaksonen, Elizabeth Watkin

**Affiliations:** ^1^ Curtin Medical School Curtin University Bentley Western Australia Australia; ^2^ The Institute for Geoscience Research, School of Earth and Planetary Sciences Curtin University Bentley Western Australia Australia; ^3^ Wesfarmers Centre of Vaccines and Infectious Diseases Telethon Kids Institute Nedlands Western Australia Australia; ^4^ Centre for Child Health Research The University of Western Australia Crawley Western Australia Australia; ^5^ John de Laeter Centre Curtin University Bentley Western Australia Australia; ^6^ Institut für Mineralogie University of Münster Münster Germany; ^7^ WA School of Mines, Minerals, Energy and Chemical Engineering Curtin University Bentley Western Australia Australia; ^8^ CSIRO Environment Perth Western Australia Australia; ^9^ School of Science Edith Cowan University Joondalup Western Australia Australia

## Abstract

Microbial attachment and biofilm formation is a ubiquitous behaviour of microorganisms and is the most crucial prerequisite of contact bioleaching. Monazite and xenotime are two commercially exploitable minerals containing rare earth elements (REEs). Bioleaching using phosphate solubilizing microorganisms is a green biotechnological approach for the extraction of REEs. In this study, microbial attachment and biofilm formation of *Klebsiella aerogenes* ATCC 13048 on the surface of these minerals were investigated using confocal laser scanning microscopy (CLSM) and scanning electron microscopy (SEM). In a batch culture system, *K. aerogenes* was able to attach and form biofilms on the surface of three phosphate minerals. The microscopy records showed three distinctive stages of biofilm development for *K. aerogenes* commencing with initial attachment to the surface occurring in the first minutes of microbial inoculation. This was followed by colonization of the surface and formation of a mature biofilm as the second distinguishable stage, with progression to dispersion as the final stage. The biofilm had a thin‐layer structure. The colonization and biofilm formation were localized toward physical surface imperfections such as cracks, pits, grooves and dents. In comparison to monazite and xenotime crystals, a higher proportion of the surface of the high‐grade monazite ore was covered by biofilm which could be due to its higher surface roughness. No selective attachment or colonization toward specific mineralogy or chemical composition of the minerals was detected. Finally, in contrast to abiotic leaching of control samples, microbial activity resulted in extensive microbial erosion on the high‐grade monazite ore.

## Graphical abstract

*Klebsiella aerogenes* form thin‐layer biofilm on monazite and xenotime. The colonization and biofilm formation were localized toward physical surface imperfections such as cracks, pits, grooves and dents. No selective attachment or colonization toward specific mineralogy or chemical composition of the minerals was detected. Finally, in contrast to abiotic leaching of control samples, microbial activity resulted in extensive microbial erosion on the high‐grade monazite ore.

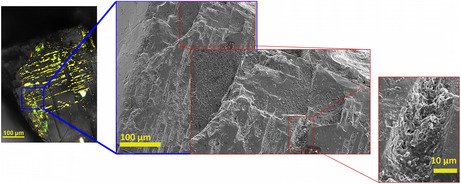

## INTRODUCTION

The depletion of high‐grade mineral resources, and high energy consumption and environmental issues of traditional mining processes have led the mining industry to search for alternative ore processing strategies (Brandl, 2001; Kaksonen et al., 2018). Bioleaching, the dissolution of minerals and mobilization of elements from insoluble ores, concentrates or wastes through biological activity, has been used at a commercial scale for the extraction of uranium and metals such as copper, cobalt, zinc, nickel, and pre‐treatment of refractory sulfidic gold ores. More recently, it has been explored for the extraction of rare earth elements (REEs) (Brandl, 2001; Fathollahzadeh et al., 2019; Kaksonen et al., 2017).

The REEs are 17 elements in the periodic table of elements, including scandium, yttrium, and 15 elements of the lanthanide series, which have many applications in the current and future technologies (Balaram, 2019). Conventional pyro‐ and hydrometallurgical REEs extraction and refining processes use high temperatures and concentrated alkalis (sodium hydroxide) or acids (sulfuric acid). Large quantities of toxic waste containing radionuclides (thorium, uranium), hydrogen fluoride, hyperacidic, or hyperalkaline wastewater, dust and associated pollutants can be produced, which results in significant environmental consequences, greenhouse gas emissions and large carbon footprint (Gwenzi et al., 2018; Haque et al., 2014).

Previous studies on monazite, an important commercially exploitable phosphate mineral containing light REEs (La, Ce, Pr, Nd), suggest bioleaching as a potential green approach for REEs extraction (Corbett et al., 2018; Fathollahzadeh, Hackett, et al., 2018). Indigenous microbial communities, acidophilic iron and sulfur oxidizing bacteria, and phosphate solubilizing microorganisms (PSM) were used as the bioleaching agents (Corbett et al., 2018; Fathollahzadeh, Hackett, et al., 2018). Amongst the tested phosphate solubilizing bacteria, *Klebsiella aerogenes*, formerly known as *Enterobacter aerogenes*, had a very high phosphate dissolution efficiency from tricalcium phosphate at 43%, and the highest REE bioleaching performance from monazite at 2 (Corbett et al., 2017). Further studies showed adding *K. aerogenes* to the native microbial community of monazite significantly promoted the REE extraction to 20 mg/L through syntrophic effects (Corbett et al., 2018). In our previous studies, the changes in pH, phosphate concentration, and released REE has been reported. A 4–8 mg/L bioleaching yield was reported for *K. aerogenes*. Furthermore, through a synergic effect, 3 days of bioleaching with *K. aerogenes* preconditioned the system for *Acidithiobacillus ferrooxidans* growth, and resulted in the highest reported REE dissolution from monazite at 40 mg/L (Corbett et al., 2017, 2018; Fathollahzadeh, Becker, et al., 2018; Fathollahzadeh, Hackett, et al., 2018). Fathollahzadeh, Becker, et al. (2018) and Corbett et al. (2018) also reported evidence of both fungal and bacterial attachment to the monazite ore. However, the process of microbial attachment and biofilm formation on the surface of these minerals is not understood.

Microbial attachment to surfaces and biofilm formation are universal phenomena of microbial life (Wu et al., 2018). In a bioleaching environment, attachment and subsequent biofilm formation have been shown to be vital prerequisites of bioleaching and result in higher bioleaching efficiency (Vera et al., 2013; Zhang et al., 2016). A mechanism of bioleaching monazite by phosphate‐solubilizing microorganisms has been proposed by Fathollahzadeh, Becker, et al. (2018) and Fathollahzadeh et al., 2019, who suggested microbial attachment to the mineral is essential. The research demonstrated that the leaching efficiencies in the absence of microbial contact were significantly lower than when microorganisms were in contact with the mineral, signifying the importance of microbial contact in comparison to non‐contact leaching (Fathollahzadeh et al., 2019; Fathollahzadeh, Becker, et al., 2018). Many studies have showed iron and sulfur oxidizing microorganisms are capable of colonizing the surface of sulfide minerals and form a biofilm (Nkulu et al., 2015; Vera et al., 2013; Zhang et al., 2016). Colonization of a sulfide mineral surfaces by these microorganisms is favoured on and around surface areas with physical imperfection or lower degrees of crystallization. Furthermore, microbial activity resulted in more severe changes on the surface and subsurface of sulfide minerals compared to abiotic controls (Nkulu et al., 2015; Vera et al., 2013; Zhang et al., 2016). With detailed evidence on the bioleaching of sulfide minerals and microbial biofilm formation on their surface, it is reasonable to hypothesise a similar mechanism of action for phosphate minerals. Nevertheless, these subjects have not been studied for phosphate minerals. In this study, microbial attachment and biofilm formation of *K. aerogenes*, localization of the biofilm formation toward physical or chemical properties of the surface, and microbial mediated changes on the surface of monazite and xenotime samples were investigated using advanced microscopy techniques.

## EXPERIMENTAL PROCEDURES

### Minerals and their characterization

A high‐grade monazite ore was provided by Lynas Rare Earths, Australia, and was sized into 50–200 μm. The sample was gamma radiated at 50 kGy for 11 h (ChemCentre) to inactivate any indigenous microorganisms. A monazite and a xenotime crystal were also used. The mineralogical characterization and composition analysis of these samples were undertaken by using X‐ray diffraction (XRD), inductively coupled plasma mass spectrometry (ICP‐MS), and TESCAN integrated mineralogy analyser (TIMA).

For the XRD‐phase identification, the high‐grade monazite ore was micronized and front‐loaded into a specimen holder, and diffraction data was collected using a Bruker D8 Discover diffractometer with Ni‐filtered Cu Kα radiation (40 kV, 40 mA) over the range 7–120° 2θ, with a step size of 0.015°. Phase identification was carried out in Bruker EVA 5.2 using the COD database. TIMA analyses were conducted at 25 kV accelerating voltage, in dot mapping mode at an analytical resolution of 3 microns for back‐scattered electron detector (BSE) acquisition and 9 microns for energy‐dispersive X‐ray spectroscopy (EDS) point spectroscopy analyses. Post‐processing of the acquired data was performed using TIMA software v. 2.1.1 (TESCAN). The laser ablation ICP‐MS analysis was conducted by Bureau Veritas following their guidelines (https://www.bureauveritas.com.au/laser‐ablation‐icp‐ms).

### Microorganism

*Klebsiella aerogenes* ATCC 13048 (formerly known as *Enterobacter aerogenes* ATCC 13048) was maintained on nutrient agar (Sigma) and prior to the experiments, was transferred into 100 mL National Botanical Research Institute's Phosphate medium (NBRIP: 5 g/L MgCl_2_(H_2_O)_6_, 0.25 g/L MgSO_4_(H_2_O)_7_, 0.2 g/L KCl, 0.1 g/L NH_4_SO_4_, 1 g/L KH_2_PO_4_, 1 g/L K_2_HPO_4_, 30 g/L glucose, pH 6.2 ± 0.4; Nautiyal, 1999) and incubated at 30°C, under aerobic conditions at 120 rpm for 3–5 days.

### Cell enumeration by flow cytometry

Absolute bacterial cell numbers were determined using flow cytometry. Bacterial cells were fixed in glutaraldehyde solution (final concentration of 2%; Sigma) and assessed in log‐fold dilution series in NBRIP media. Bacteria were suspended in a final volume of 1 mL of filter sterilized (0.22 μ, Millipore) NBRIP medium and stained with 5 μL of 1.67 mM solution SYTO‐9 (Invitrogen) per one millilitre of bacterial suspension for 20 min at room temperature, in the dark.

Samples were kept on ice in the dark before acquisition. Bacterial numbers were acquired on the four‐laser configuration (405, 488, 561 and 638 nm) Attune NxT Acoustic Focusing Flow Cytometer (Invitrogen). The cytometer was set to acquire 50 μL of sample at an acquisition rate of 25 μL/min. The threshold was set to the lowest SSC‐H value (0.1 × 10^3^) to determine true bacterial events from that of electronic noise, using negative controls that contained NBRIP alone, unstained bacteria in NBRIP or NBRIP with SYTO‐9 (Figure S5).

A primary gate was used to identify bacteria through 488 nm SSC profiles and the fluorescence signal of SYTO‐9. Data were exported as FCS3.0 files and analysed in FlowJo v10.7 (BD Biosciences). Bacterial SYTO‐9+ events were divided by the total acquisition volume to obtain the concentration of cells/mL. This value was used to determine the volume of stock bacterial culture needed to achieve a final concentration of 1 × 10^7^ cell/mL in the test flasks.

### Evaluation of biofilm formation

Confocal laser scanning microscopy (CLSM) and scanning electron microscopy (SEM) were used to monitor initial attachment and colonization of the minerals surface, localization of biofilm formation, and microbial mediated changes on the surface of the minerals by *K. aerogenes* (Tuck et al., 2021).

The bioleaching setting was designed following our previous studies (Corbett et al., 2018; Fathollahzadeh, Becker, et al., 2018; Fathollahzadeh, Hackett, et al., 2018). The high‐grade monazite ore, monazite‐muscovite crystal, and xenotime crystal (0.5–2 mm) samples were subjected to bioleaching by *K. aerogenes* in NBRIP medium using these mineral samples as the sole source of phosphate. Ore and crystal samples were fixed on pre‐autoclaved standard microscope slides (glass, Westlab) using an epoxy adhesive that provides glass‐like transparent curing (E6000 Multipurpose Adhesive). The slides were sterilized by submerging them in 70% ethanol for 10 min and then exposed to UV for 1 h on each side. The slides were then placed inside flasks containing 100 mL phosphate free NBRIP medium with an initial pH of 6.2 ± 0.4 and an initial inoculum size of ~1 × 10^7^ cells/mL pre‐enumerated using flow cytometry. The flasks were incubated at 30°C. In previous studies shaking speeds of 120 and 130 rpm were used (Corbett et al., 2018; Fathollahzadeh, Becker, et al., 2018; Fathollahzadeh, Hackett, et al., 2018). However, 120 or 130 rpm was not enough to provide enough oxygen when the slides were used in the system. Hence, 140 rpm was used as the shaking speed. The slides were removed from the media at 4, 8, 16, 24 h, and 2, 3, 5, 8, 11 and 14 days.

Immediately after sample collection, the samples were washed with 25 mL sterile NBRIP media to remove planktonic and loosely attached cells from the surface of the samples. This step was repeated five times. A silicon cavity (Proscitech) was fixed on the slides and filled with 500 μL of staining mixture containing Hoechst 33342 (1 μM) and DiTO‐1 (1 μM) in phosphate and glucose‐free NBRIP media, incubated in the dark at ambient temperature for a minimum of 20 min. The samples were washed with 25 mL sterile NBRIP medium to remove the staining solution (Li et al., 2016). These two fluorochromes were used to maximize cell visualization. Hoechst 33342 (AATBio) is a membrane permeant dye that stains any DNA molecule inside and outside the cells, genomic and extracellular DNA (eDNA), respectively. DiTO‐1 (AATBio) is a membrane impermeant dye that only stains the DNA molecules external to the cell walls of living cells (eDNA) and the genomic DNA of the dead cells, or lives cells with compromised membrane.

The samples were air‐dried at room temperature. Then 500 μL glycerol was added to the cavity; the cover slide was then sealed with clear nail polish. The samples were kept in the dark and cold (4°C) until CLSM imaging. Chemical leaching was tested using sterile Milli‐Q water and sterile NBRIP medium at three different pH values (pH 6, 5, and 4) and was used as a control to study any changes in the autofluorescence of the samples. The samples were imaged using a Nikon A1+ point scanning confocal microscope with NIS elements software (Nikon Instruments). Hoechst 33342 was excited at 405 nm laser and detected through 450/50 filter. DiTO‐1 was excited at 457 nm laser and detected through 525/50 filter. The colonization and biofilm formation was studied using 10× and 20× objectives.

### Live imaging

Bioleached mineral samples were studied using live mode CLSM imaging. Bioleaching was conducted in conical flasks with phosphate free NBRIP medium at a 1% slurry of monazite as the sole phosphate source and an inoculum of ~1 × 10^7^ cells/mL. Samples of the high‐grade monazite ore were taken at 4, 8, 16, 24 h, and 2, 3, 5, 8, 11 and 14 days. To further evaluate the changes in the biofilm after the initial dispersion stage sampling was continued to day 70 (at days 20, 25, 30, 35, 40, 45, 50, 60, and 70). The samples were collected in microtubes and allowed to settle at room temperature for 1 min. To remove the planktonic and unattached cells from sediments, the supernatant was discarded, 1 mL glucose and phosphate‐free NBRIP medium was added and the microtubes mixed by gentle inversion, the ore was allowed to settle and the supernatant was discarded. This was repeated 5 times. The staining procedure followed the same methodology as above using NBRIP with glucose to support the metabolic activity of the cells. After staining, the mixture was transferred to a μ‐Slide 2 Well Glass Bottom microscope slide (ibidi).

CLSM images were collected in Z‐stack mode with 3.5–7 micron intervals (×10 and ×20 objective lenses) or ≤1 micron intervals (×40 and ×100 objective lenses). The images were analysed in Nikon NIS elements software and were visualized in maximum intensity projections mode. The 3D structure of the samples was created and recorded. In the case of high‐grade monazite samples the acquired Z‐stack CLSM images were reanalysed in Imaris v 9.7 (Oxford Instruments) to detect selective attachment of the cells to specific surface properties. The surface of the monazite samples was reconstructed using the “Surface” tool and the cells were modelled using “Spots” tool to provide a better 3D view of both cells and the surface. To assess autofluorescence of the samples, abiotic leaching was conducted in NBRIP media and Milli‐Q water at three different pH values: 4, 5 and 7.

### Fluorescent microscopy

Live fluorescent microscopy was used to study bacterial attachment 10 min after inoculation. *K. aerogenes* pre‐stained with Hoechst 33342 was added to a high‐grade monazite ore sample in 500 μL NBRIP medium in a μ‐Slide 2 Well Glass Bottom microscope slide. Hoechst 33342 was visualized using WB: blue excitation (wide band) filter (Ex 465/15, FT 500, LP 515).

### SEM–EDS

After obtaining the CLSM micrographs of the immobilized monazite and xenotime samples, the same samples were used for SEM imaging and EDS analysis. The samples were subjected to carbon (10 nm) and platinum (3 nm) coating (CMCA) for high‐resolution imaging and studied on TESCAN Clara FESEM microscope using TESCAN Essence software (John de Laeter Centre [JdLC], Curtin University) at an operating voltage of 5 or 10 kV and beam intensity of 300 pA. Images were taken using secondary electrons (SE), or a dual detector using SE and backscattered electrons (SE/BSE). For SE an accelerating voltage of ≥5 kV and beam intensity of 300 pA were used, for EDS analyses an accelerating voltage of ≥15 kV and beam intensity of 1 nA were used, and for SE/BSE imaging either of the two settings were used.

### Focused ion beam‐ scanning electron microscopy (FIB‐SEM)

Cross‐sectional analysis of the biofilm on high‐grade monazite ore was conducted using a Tescan Lyra3 FIB‐SEM located at JdLC, Curtin University, which has a monoisotopic ^69^Ga^+^ liquid metal ion source. This dual‐beam system can achieve an imaging resolution of <2.5 nm (at 30 kV) for the Cobra FIB column, and 1 nm (at 30 kV) for the SEM column. A 1 μm thick platinum (Pt) layer was deposited through electron beam and ion beam to protect the surface profile of the region to be analysed. Electron beam deposition is a non‐destructive process which provides the best protection of the biofilm. Subsequently a 30 kV ion beam was employed to cross‐section the region of interest by sputtering approximately 10 μm of material away and progressively polishing the cross‐section with lower currents.

## RESULTS

### Mineral characteristics

The mineral characterization analyses of the high‐grade monazite ore (Figures S1 and S2 and Tables S1–S4) indicated that monazite was the main mineral group in this sample. Crandallite, florencite, beryl, quartz, goethite, haematite/magnetite and several other minerals were also present in this sample. Moreover, TIMA mineral mapping showed difference in the mineralogy and chemical composition between different grains of the high‐grade monazite. Due to the high mineral complexity, some grains and regions were rich in some elements specifically Al, Ca, Si, Fe, and REE, while others contained less or none of these elements (Figures S1 and S14).

The monazite‐muscovite ore mainly consisted of two major mineral plates, the monazite plates rich in REEs‐phosphate and aluminium silicate plates determined as muscovite using TIMA analysis (Figure S3). This sample also contained florencite, haematite and several other minerals.

Xenotime crystal showed relatively homogenous mineralogy (Figure S4), mainly containing xenotime group of minerals.

### Biofilm formation

No or negligible autofluorescence due to abiotic leaching was detected using CLSM, signifying no or negligible effect of mineral autofluorescence on the recorded fluorescence of the bioleaching samples (Figure S6). Therefore, the recorded fluorescence of the bioleaching samples was only representative of microorganisms.

**FIGURE 1:**
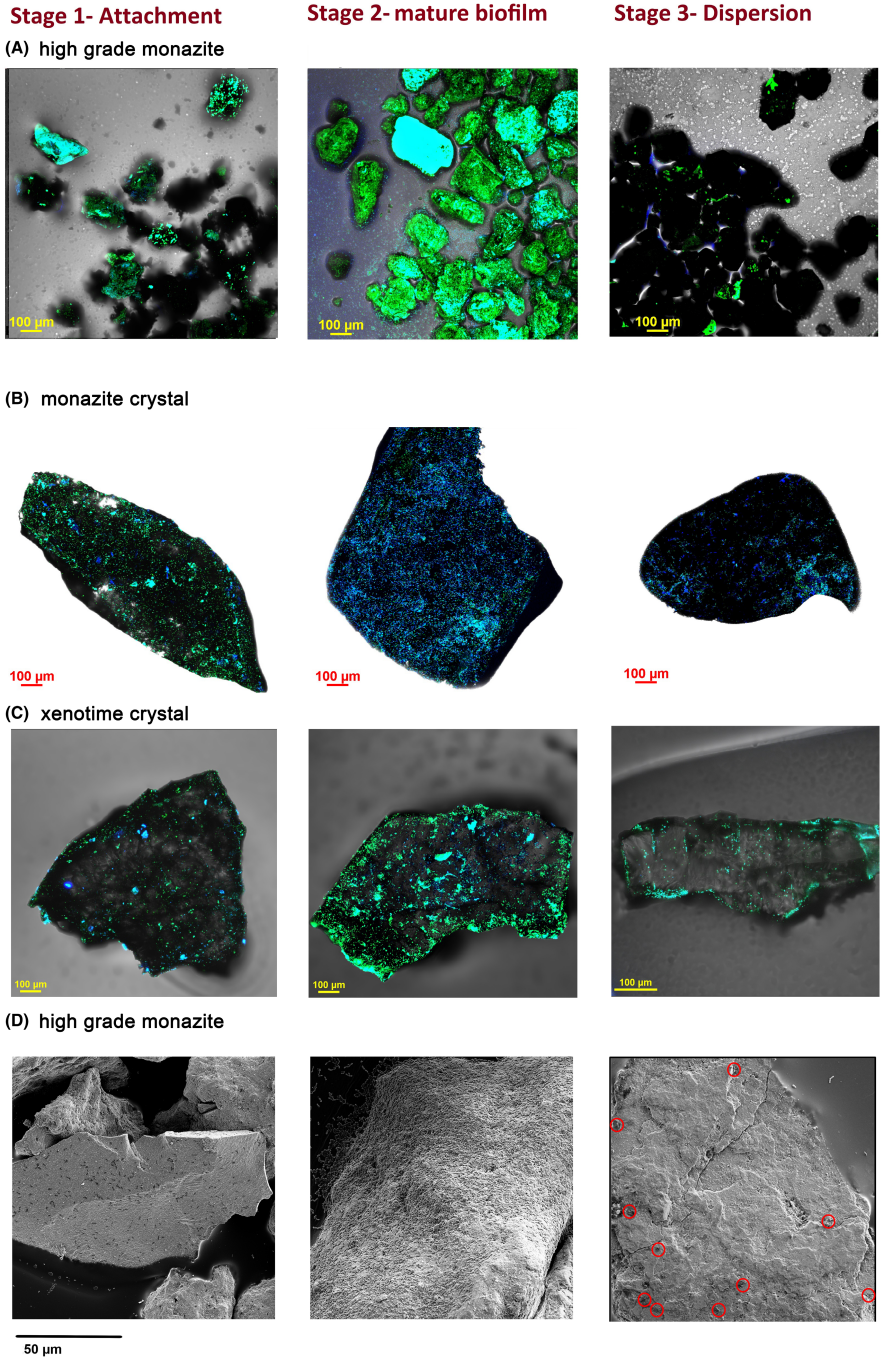
Visualization of *Klebsiella aerogenes* biofilm on the surface of high‐grade monazite ore (A, D), monazite‐muscovite crystals (B) and xenotime crystals (C). CLSM images are the merged images of Hoechst 33342 (blue) and DiTO‐1 (green) to maximize microbial cell visualization, and transmitted light channels. The image shows the maximum fluorescent intensity of the acquired Z‐stack images where blue, green or cyan colours on the surface of the ore grains (the dark grains) are representative of the microbial biofilm, the red circles in secondary electron SEM images (panel D) indicate on the small number of bacteria on the surface.

*Klebsiella aerogenes* was able to use high‐grade monazite ore as the sole source of phosphate and the planktonic cell number increased from ~1 × 10^7^ to ~1 × 10^9^ cell/mL in 2 to 3 days (data not shown). It was also capable of growing on both monazite and xenotime crystals. *K. aerogenes* was capable of attachment to the surface of high‐grade monazite ore (Figure 1A,D; Figures S7 and S10), monazite‐muscovite crystals (Figure 1B; Figure S8), and xenotime crystals (Figure 1C; Figure S9). Biofilm formation and development occurred over three stages, initial attachment during the first 8 h after inoculation, colonization and maturation from day 1 to day 8, and dispersion at day 11 onward.

Initial attachment occurred very early after bacterial inoculation. Ten minutes after inoculation, *K. aerogenes* cells were already attached to the surface of the high‐grade monazite (data not shown). The CLSM records 4 and 8 h after bioleaching showed a high extent of attachment to some of the ore grains. This initial attachment led to the second stage of biofilm formation, the colonization of the surfaces (16–24 h) and the formation of a mature biofilm over the following days (day 2 to day 8) (Figures S7 and S10).

**FIGURE 2:**
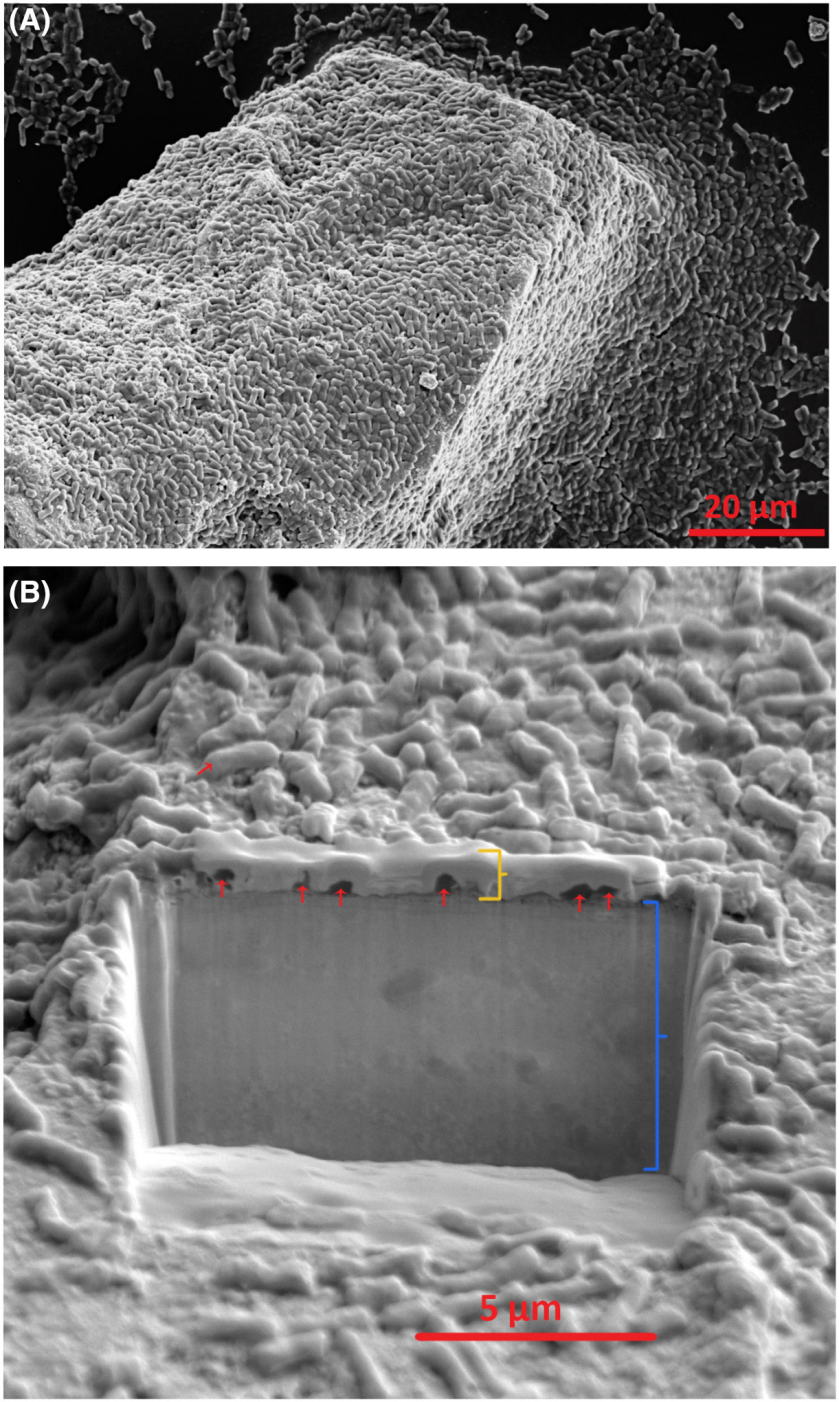
SEM micrographs of (A) high‐grade monazite grain covered *in Klebsiella aerogenes* mature biofilm, and (B) a FIB cross‐section as the surface of the grain revealing a ~500 nm thick biofilm on the surface of the mineral. A 1‐μm thick Pt protection layer was deposited on the region of interest before the FIB cut to keep the surface profile of the region. Red arrows indicate bacteria, yellow bracket indicate the 1‐μm thick Pt protection layer, and blue bracket shows the subsurface of high‐grade monazite in the cross section.

From day 11, CLSM and SEM records indicated a reduction in the biofilm, marking the third stage, dispersion, with a significant proportion of the sessile population detaching from the biofilm as shown by a dramatic decrease of the biofilm‐related fluorescence on the surface of the ore grains (Figure S7). At day 14, while very few cells were attached to the surface for some of the ore grains, some other grains partially harboured active biofilm. SEM confirmed these findings showing cell numbers attached to the surface of the ore were increasing from early attachment to colonization and maturation stage, and then was decreasing by day 14 (Figure S10). The live microscopy records of the high‐grade monazite ore continued to 70 days after inoculation and showed signs of active re‐colonization and dispersion after the first dispersion at days 11 and 14 (Figure S11). A similar pattern was observed for the other two mineral samples, monazite‐muscovite (Figure S8) and xenotime (Figure S9) crystals. Moreover, the SEM and CLSM micrographs showed that the biofilm structure was a thin‐layer biofilm (Figure 2; Figure S12).

### Localization of attachment and biofilm formation to physical imperfections

**FIGURE 3:**
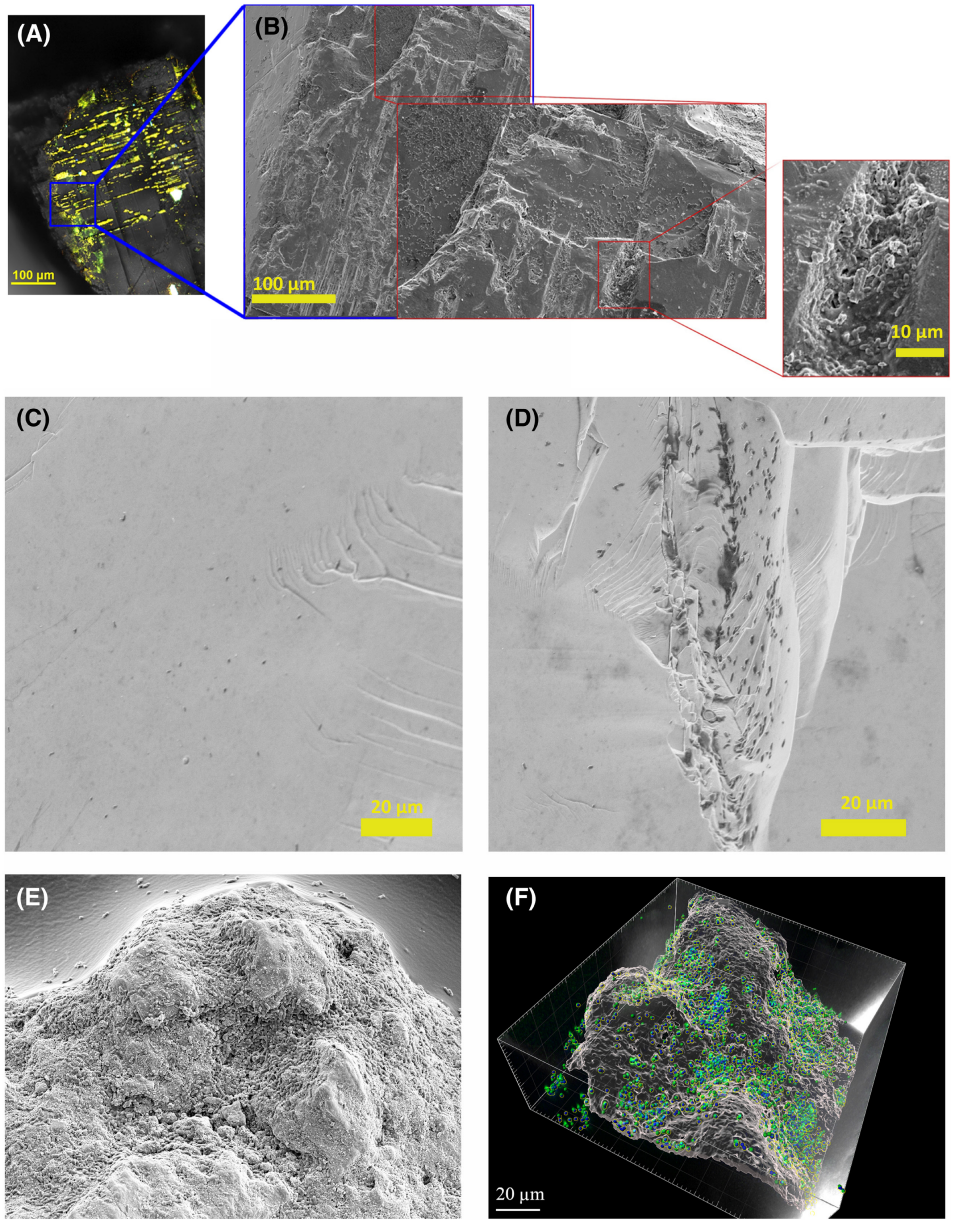
Localization of *Klebsiella aerogenes* biofilm on the surface of xenotime crystal visualized by CLSM (A) and secondary electron SEM (B), monazite‐muscovite crystal visualized by SEM (C, D), and high‐grade monazite ore by SEM (E) and CLSM (F). In panel “A” the golden area is representative of bacterial biofilm. In panel “F”, the surface and the bacteria are reconstructed from CLSM Z‐stack images using Imaris software. Bacteria are the blue/green spheres (blue for genomic‐DNA, green for eDNA) and the surface is semi‐transparent white area.

Attachment, further localization and the resultant formation of biofilm of *K. aerogenes* were directed toward specific areas on the minerals' surface, namely physical imperfections (cracks, grooves, dents, scratches, holes, pits, edges, etc.) on the surface of high‐grade monazite ore (Figure 3E,F), monazite‐muscovite (Figure 3C,D; Figure S13), and xenotime crystals (Figure 3A,B; Figure S14). SEM micrographs showed that while the reasonably flat surface of xenotime or monazite‐muscovite crystals could harbour bacterial cells, the greatest intensity of biofilm formation was on and around the surface imperfections. A similar attraction and a higher density of microbial biofilm in areas with physical imperfections were also observed for high‐grade monazite ore (Figure 3E,F).

### Localization of attachment and biofilm formation to specific mineralogy or chemical composition

**FIGURE 4:**
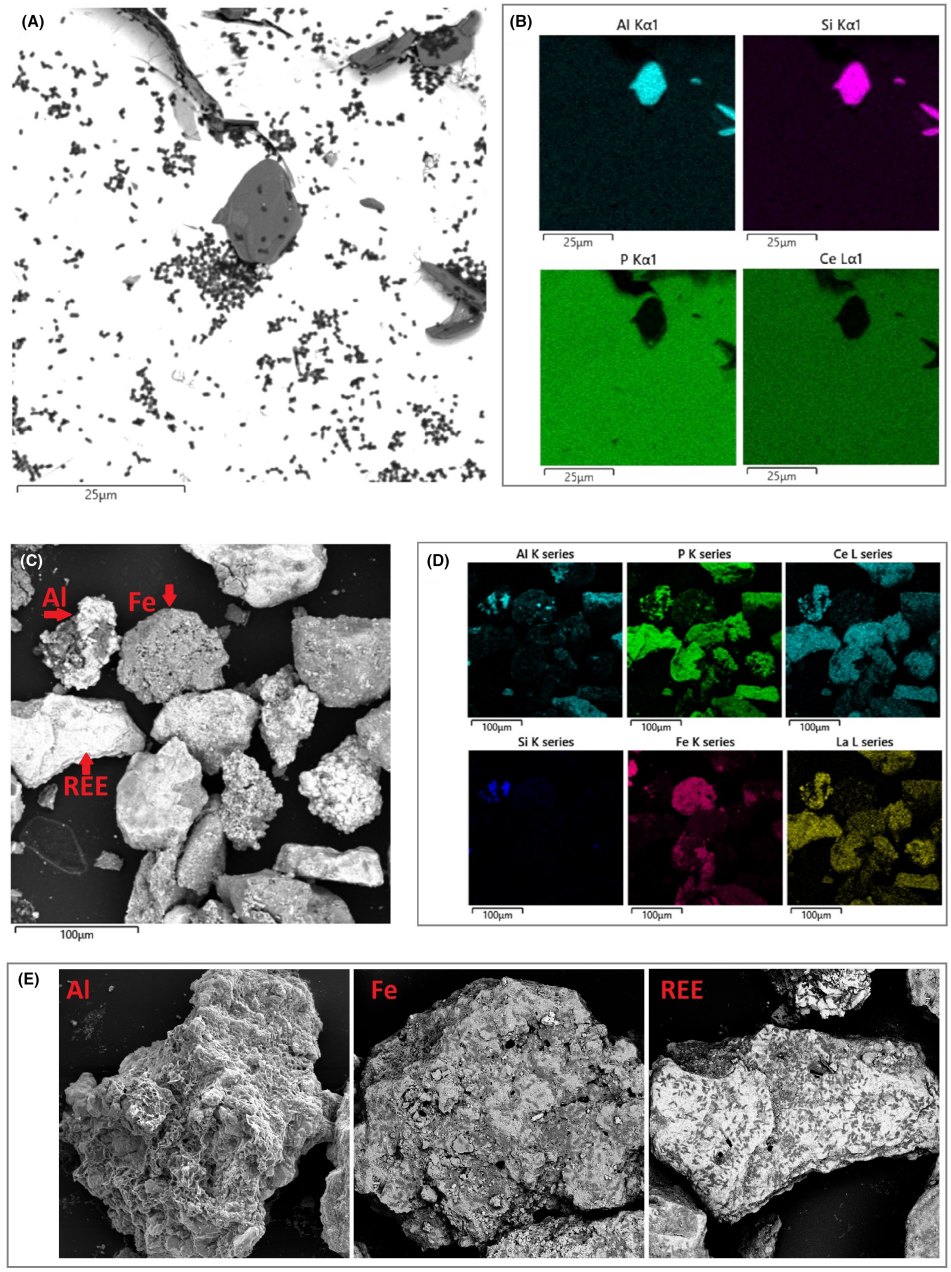
*Klebsiella aerogenes* biofilm on monazite‐muscovite crystals were monazite are the white regions, and muscovite plates are the dark plates (A), and high‐grade monazite ore (B). *K. aerogenes* cells are the small black/grey bacilli shapes on the surface. The SEM (SE/BSE dual detector imaging) micrographs (A, C, E) were obtained with backscattered electron imaging; hence heavier elements depicted brighter and light elements are depicted darker. The SEM–EDS elemental map analysis shows chemical composition of different mineral plates and grains in the samples (B & D). The black regions in the SEM–EDS maps (B & D) represent absence of an element of interest and the coloured area represent the areas on the surface where the related EDS signal was detected for that specific element.

Biofilm formation was localized on and around physical imperfections; however, no specific localization based on the differences in chemical or mineral composition was observed. *K. aerogenes* developed small micro‐colonies on both monazite (Figure 4A, white regions in the SEM micrograph) and muscovite (Figure 4A, dark regions in the SEM micrograph) plates of monazite‐muscovite crystals. In the case of high‐grade monazite ore, a biofilm formed on all ore high‐grade grains, independent of the mineral or chemical composition such as aluminium rich, iron rich or rare earth rich regions (Figure 4C,E; Figures S15 and S16).

### Changes on the surface and subsurface of the monazite minerals due to microbial activity

**FIGURE 5:**
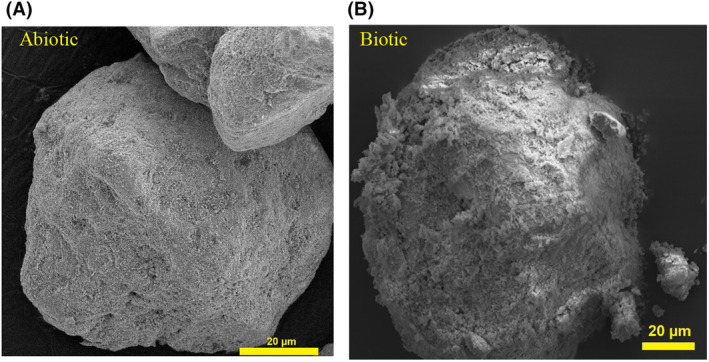
Visualization of the surface of high‐grade monazite ore after 14 days of abiotic leaching (A) and bioleaching in the presence of *Klebsiella aerogenes* (B).

SEM was used to investigate changes to the mineral surface as a result of microbial activity since CLSM was not a suitable tool to study such changes in detail (data not shown). After 2 weeks of bacterial activity, some of the grains of high‐grade monazite ore samples showed dramatic surface erosion due to microbial activity; however, such erosion patterns were not observed in the abiotic control samples (Figure 5).

## DISCUSSION

Biofilm is a universal attribute and a prevalent form of microbial life in natural ecosystems for most microorganisms, if not all (Rumbaugh & Sauer, 2020). Biofilm formation is of value in bioleaching‐based technology. Of the three proven mechanisms for bioleaching of sulfide minerals, contact, non‐contact and cooperative models (Kaksonen et al., 2018; Natarajan, 2018; Panda et al., 2015), two mechanisms (contact and cooperative) require biofilm formation. Fathollahzadeh, Becker, et al. (2018) conceptualized the bioleaching of phosphate minerals in the same three mechanisms and showed that the leaching efficiencies in the absence of contact to minerals were significantly lower than in contact with minerals, which signifies the importance of microbial contact in comparison to non‐contact leaching (Fathollahzadeh et al., 2019; Fathollahzadeh, Becker, et al., 2018). The current study provides evidence to validate the most critical aspect of REE contact leaching, the formation of microbial biofilm on the surface of the REE minerals.

Similar to other in vivo or in vitro studies of microbial biofilm formation, the microscopy data confirmed the three main stages of biofilm formation for *K. aerogenes* on the surface of phosphate minerals. The first step in forming a biofilm involves introduction of the bacteria to a surface (Donlan, 2002; Kostakioti et al., 2013). The initial attachment of *K. aerogenes* started very early after exposure to the high‐grade monazite ore. This early phase of initial attachment is a dynamic and reversible process which is in response to nutrient availability, hydrodynamic or repulsive forces (Kostakioti et al., 2013). However, given enough time, the early attachment leads to colonization and further development of mature biofilm, the second major distinguishable stage of biofilm development. This occurs when the cells are ready to commit to a biofilm lifestyle which requires irreversible attachment to the surface (Kostakioti et al., 2013; Marshall et al., 1971). The colonization continued further until almost the whole available surface of the high‐grade monazite ore and a notable area on the monazite or xenotime crystals were covered with a bacterial biofilm which marks the maturation stage. The maturation stage favours a higher expression of factors favouring sessile state such as those associated with extracellular polymeric substances (EPS) production (Bellenberg et al., 2015; Kostakioti et al., 2013; Toyofuku et al., 2016). Although biofilm architecture is continuously changing as a result of the external and internal factors, it finds its most complex and stable form at the maturation stage. The SEM records of *K. aerogenes* mature biofilm demonstrated that the formed biofilm on the surface of phosphate minerals was a thin‐layer structure (Figure 2). The complexity of the biofilms' 3D structure varies for different microorganisms and environmental conditions with single‐species biofilm of some bacteria such as *Pseudomonas aeruginosa* capable of forming multi‐layer complex structures known as the mushroom‐shaped biofilm (Kostakioti et al., 2013). One reason for the simple structure of *K. aerogenes* biofilm could be due to a relatively high fluid shear force generated from shaking at 140 rpm which can affect the structure, density and attachment strength, or even metabolic activity of a biofilm (Stoodley et al., 2002).

The final stage of a biofilm development cycle is dispersion (Rumbaugh & Sauer, 2020). Detachment is dynamic and heavily affected by the external and internal factors such as pH, temperature, and chemotaxis inside the biofilm matrix (Donlan, 2002; Rumbaugh & Sauer, 2020). However, at some point after the maturation stages a considerable proportion of the sessile subpopulation leaves the biofilm lifestyle. For *K. aerogenes* this process was initially observed at day 11 onward when a notable decrease in the biofilm was recorded in both CLSM and SEM records in contrast to the maturation phase (days 1–8). Dispersion rarely involves the entire biofilm and occurs through passive or active mechanisms (Rumbaugh & Sauer, 2020). The fluid shear force, erosion and sloughing by fluid frictional force, and abrasion due to collisions of the biofilm with floating mineral particles can lead to passive detachment of the *K. aerogenes* biofilm (Chiume et al., 2012; Donlan, 2002; Rumbaugh & Sauer, 2020). Moreover, live microscopy performed between days 20 and 70 (Figure S11) show signs of active re‐colonization and dispersion of the ore grains after the initial dispersion observed at day 11 onward indicating an active seeding‐dispersal mechanism (Rumbaugh & Sauer, 2020) for *K. aerogenes*.

A critical question in biofilm studies is regarding the localization of the microbial biofilm. Microbial colonization of a surface and biofilm formation are not entirely random processes and can be influenced by topography and chemical or physical properties of a surface (Bellenberg et al., 2015; Wu et al., 2018; Zhang et al., 2016). In bioleaching settings, biofilm formation is predominant on the sites with higher surface roughness, physical imperfections, and surfaces with lower degrees of crystallization (Sanhueza et al., 1999; Vera et al., 2013; Zhang et al., 2016). The formed biofilm on xenotime crystal (Figure 3; Figure S14) shows the localization of a *K. aerogenes* biofilm on and around physical imperfections of the surface (dents, grooves and cracks). The CLSM micrograph (highlighted in gold –Figure 3A, Figure S14) represents the biofilm and is well matched with the physical imperfections. The SEM of the same surface demonstrated the number of attached cells on the reasonably flat surfaces with minimum topographical imperfections was lower than on and around the physical defect sites of the surface where some micro‐colonies were formed. A similar pattern was observed for the monazite‐muscovite crystals, (Figure 3; Figure S13). The same was also observed for high‐grade monazite ore in which both SEM (Figure 3E) and computer‐aid reconstructed CLSM image (Figure 3F) demonstrate localization of biofilm on the grooves. It is notable that the total biofilm‐covered area versus biofilm‐free area on the monazite‐muscovite crystals or xenotime crystals was lower than that of the high‐grade monazite ore. This could be due to differences in the surface properties of the samples, including roughness, imperfections and crystallinity degree as the high‐grade monazite ore has a higher surface roughness compared to the other two mineral samples.

Previous research on bioleaching biofilms (Bellenberg et al., 2015; Vera et al., 2013; Zhang et al., 2014, 2015) showed that *Acidithiobacillus ferrooxidans* selectively attaches and forms biofilms predominantly on and around physical and topographic imperfections sites on the surface of the minerals (such as pits, pores, holes, cavities, cracks and scratches, dents, steps and layers), crystal properties (degree of crystallization, crystal defects, grain boundaries and crystallographic orientation), and areas with different surface charge imbalances or hydrophobic/hydrophilic areas.

Easier attachment due to more favourable surface properties such as energy, charge imbalance, hydrophobicity–hydrophilicity (Zhang et al., 2016), and degree of crystallinity (Sanhueza et al., 1999) are suggested as reasons for selective attachment and biofilm formation on and around the topographical and physical imperfections by some microorganism. Moreover, such locations on the surface provide a higher nutrient availability due to these sites being potential active dissolution regions on the surface, and also provides safety from the sheer fluid force (Bellenberg et al., 2015). Furthermore, the transitory chemotactic attraction of electrically charged microbial cells toward dissolution sites may lead to the formation of anode and cathode regions on the minerals' surface, which drives the dissolution process further. In such a system, the EPS of a biofilm fills the space between the microbial cells and the surface of the mineral as the matrix interface of the leaching reaction (Zhang et al., 2016). The reconstructed 3D models from recorded CLSM micrographs during the dispersion stage (Video S1) show that the developed biofilm inside some visible topographical imperfections stands still at the dispersion stage. Therefore, the topographical imperfections also provide protection against passive detachment due to weaker impacts of fluid shear forces or collisions with mineral particles floating around (Bellenberg et al., 2015; Chiume et al., 2012).

Another critical question of the current study was to see if *K. aerogenes* shows selective attachment toward specific mineralogy or chemical composition on the surface. In addition to physical properties, it has been shown that specific mineralogy or chemical composition of a surface, ionic strength, and presence of certain ions can influence the interaction, attachment, and biofilm development behaviour of microorganisms (Bellenberg et al., 2015; Zhang et al., 2016). Figures 4A,B demonstrate *K. aerogenes* attachment and micro‐colony formation occurred on both mineral plates of monazite‐muscovite sample, the monazite plate which is rich in REEs phosphates such as cerium (Ce) and phosphate (P) and muscovite plate rich in aluminium (Al) and silicate (Si). The same pattern was observed for the high‐grade monazite sample with a much more complex mineralogy (Figures 4C–E; Figures S15 and S16). Despite having a diverse range of minerals and chemical distribution of elements on the high‐grade monazite sample, *K. aerogenes* biofilm formed on all high‐grade monazite ore grains. Xenotime crystals did not show complex mineralogy and were not used for this experiment. Therefore, unlike some of the iron and sulfur oxidizing microorganisms (Bellenberg et al., 2015), *K. aerogenes* showed no selectivity toward specific chemical or mineral composition under the tested conditions. Nevertheless, this observation cannot be generalized to other phosphate minerals or phosphate solubilizing microorganisms, since the current study is the only available study addressing this aspect of biofilm formation on such minerals and using such microorganisms.

Changing the surface topography, dissolution of some elements, and formation of secondary minerals can occur as a result of both biotic and abiotic leaching and has been reported for both sulfide (Bellenberg et al., 2015; Liu et al., 2011; Nkulu et al., 2015) and phosphate minerals (Ceci et al., 2015). The SEM images of *K. aerogenes* activity revealed that after 14 days of bioleaching, the surface of some of the high‐grade monazite ore grains subjected to microbial activity notably changed in the form of an eroded surface, while the abiotic leaching controls with similar pH did not show drastic visible topographical changes and erosion patterns (Figure 5). Fathollahzadeh, Becker, et al. (2018) reported a similar observation on MWM monazite, another sample from Lynas Corporation (Fathollahzadeh, Becker, et al., 2018).

Based on the finding of this study, it is reasonable to say in a controlled environment like bioreactor‐based bioleaching, a pre‐processing such as grinding and milling to fine particles can be done on ore or waste samples to increase the total available surface well as physical properties of the surface to promote microbial attachment and as a result leaching efficiency.

## CONCLUSION

*Klebsiella aerogenes*, as the model phosphate solubilizing microorganism, was able to colonize the surface of monazite and xenotime and form a thin‐layer biofilm. Biofilm development occurred in three distinctive stages, initial attachment, maturation and dispersion. Colonization and biofilm formation were selective toward physical imperfection such as grooves and cracks but not toward specific mineralogy or chemical composition on the surface. The selective colonization on and around the physical imperfections could be due to easier access to nutrient on and around these dissolution sites. Such topographical imperfections provided *K. aerogenes* sessile population with protection against the fluid shear forces during initial attachment of the bacterial cells and maturation of the biofilm. The biofilm formed in the grooves and cracks were also protected from passive detachment due to weaker impacts of fluid shear forces or collisions with mineral particles.

## AUTHOR CONTRIBUTIONS

**Arya van Alin:** Conceptualization (lead); data curation (lead); formal analysis (lead); investigation (lead); methodology (lead); project administration (lead); software (lead); validation (lead); visualization (lead); writing – original draft (lead); writing – review and editing (lead). **Melissa K. Corbett:** Conceptualization (equal); supervision (equal); writing – review and editing (equal). **Homayoun Fathollahzadeh:** Conceptualization (equal); methodology (equal); writing – review and editing (equal). **M. Christian Tjiam:** Methodology (equal); writing – review and editing (supporting). **William D. A. Rickard:** Formal analysis (equal); investigation (equal); methodology (equal); writing – review and editing (supporting). **Xiao Sun:** Formal analysis (equal); investigation (equal); methodology (equal); writing – review and editing (supporting). **Andrew Putnis:** Conceptualization (equal); funding acquisition (lead); methodology (equal); supervision (equal); writing – review and editing (equal). **Jacobus Eksteen:** Conceptualization (equal); funding acquisition (equal); supervision (equal). **Anna H. Kaksonen:** Supervision (equal); writing – review and editing (equal). **Elizabeth Watkin:** Conceptualization (lead); funding acquisition (equal); resources (lead); supervision (lead); writing – review and editing (equal).

## FUNDING INFORMATION

The authors acknowledge the Australian Research Council (ARC) for grant DP200103243. The authors acknowledge The Institute for Geoscience Research for financial support through TIGeR Small Grants.

## CONFLICT OF INTEREST STATEMENT

The authors declare no competing financial or non‐financial interests.

## Supporting information


Appendix S1

Video S1

## Data Availability

The authors confirm that the data supporting the findings of this study are available within the articles supplementary materials or from the corresponding author (EW) on request.
