# Supplementary material for: Biofilm formation on the surface of monazite and xenotime during bioleaching
Source: Microb Biotechnol. 2023 Jun 8;16(9):1790–802. doi: 10.1111/1751-7915.14260 (PMC10443343; doi:10.1111/1751-7915.14260)
Supplement: Supplementary file 1 — Appendix S1 [file MBT2-16-1790-s001.docx]

Supplementary material

**Biofilm formation on the surface of monazite and xenotime during bioleaching**

Arya van Alin ^1, 2^, Melissa K Corbett^1, 2^, Homayoun Fathollahzadeh^1,2^, M. Christian Tjiam^1, 3, 4^, William D.A. Rickard ^5^, Xiao Sun ^5^, Andrew Putnis^2,6^, Jacques Eksteen^7^, Anna H Kaksonen^7, 8^, Elizabeth Watkin^1, 2*§^

^1^ Curtin Medical School, Curtin University, Western Australia, Australia

^2^ The Institute for Geoscience Research, School of Earth and Planetary Sciences, Curtin University, Western Australia, Australia

^3^ Wesfarmers Centre of Vaccines and Infectious Diseases, Telethon Kids Institute, Western Australia, Australia

^4^ Centre for Child Health Research, The University of Western Australia, Western Australia, Australia

^5^ John de Laeter Centre, Curtin University, Western Australia, Australia

^6^ Institut für Mineralogie, University of Münster, Germany

^7^ WA School of Mines, Minerals, Energy and Chemical Engineering, Curtin University, Western Australia, Australia

^8^ CSIRO Land and Water, Western Australia, Australia

*Correspondence: e.watkin@ecu.edu.au (Elizabeth Watkin).

§Current address: School of Science, Edith Cowan University Western Australia, Australia

**Table S-1.** The reference chemical composition of the detected minerals in this study

| Mineral | Composition | Comments |
| --- | --- | --- |
| Monazite group | MTO₄ | Monazite Group: Where M = REE, Th, Ca, Bi; T = P, As. Monazite-(Ce) and Cheralite were used in these Quantitative X-ray Diffraction QXRD refinements. |
| Goethite | (Feₓ M₁₋ₓ)O(O H) | Goethite: Where M=Al and various other cations |
| Quartz | SiO₂ |  |
| Kihlmanite | Ce_2_TiO_2_(SiO_4_)(HCO_3_)_2_·H_2_O | Kihlmanite: Kihlmanite-(Ce) was used in these QXRD refinements. The peak identified can be overlap with other phases. Further investigation would be required for more accurate classification. |
| Xenotime | YPO_4_ | Xenotime: Xenotime-(Y) was used in these QXRD refinements. |
| Hematite group | R₂O₃ | Hematite group: Where R=Al, Cr³⁺, Fe³⁺, V³⁺. Hematite was used in these QXRD refinements. |
| Crandallite group | CaAl₃(PO₄)(PO₃OH)(OH)₆ | Crandallite group: Crandalite was used in these QXRD refinements. Crandallite and Florencite peaks overlap. Further investigation would be required for more accurate classification. |
| Alunite group | AB₆(SO₄)₄(OH)₁₂ | Alunite group: Where A=Ag, Ca, (H₃O)₂, K₂, Na₂, (NH₄)₂, Pb; B=Al, Cu²⁺, Fe³⁺. Jarosite and Florencite-(Ce) were used in these QXRD refinements. Florencite and Crandallite peaks overlaps. Further investigation would be required for more accurate classification. |


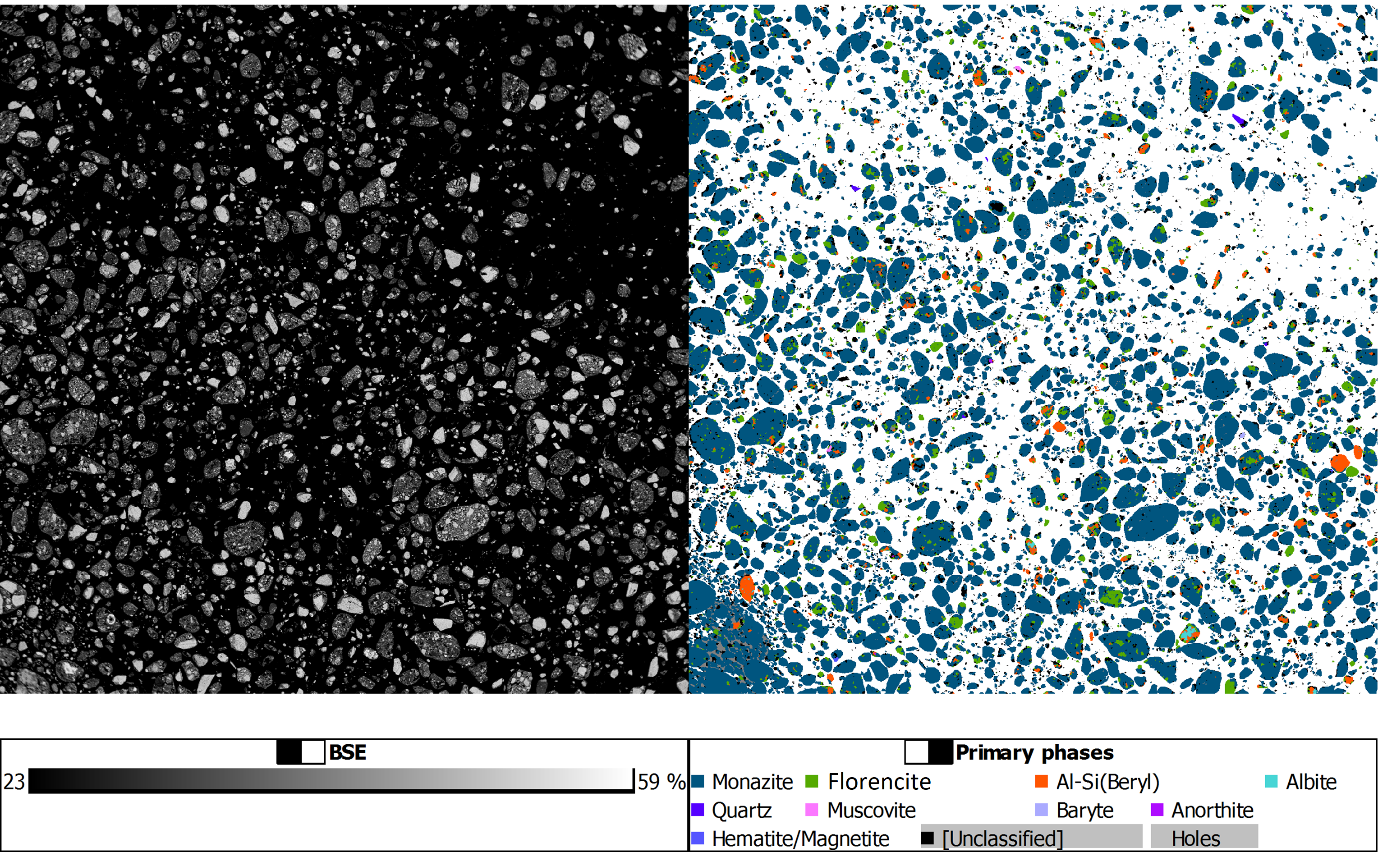


**Figure S-1.** High-grade monazite ore TESCAN Integrated Mineralogy Analyser (TIMA) mapping conducted at John de Laeter Centre, Curtin University.


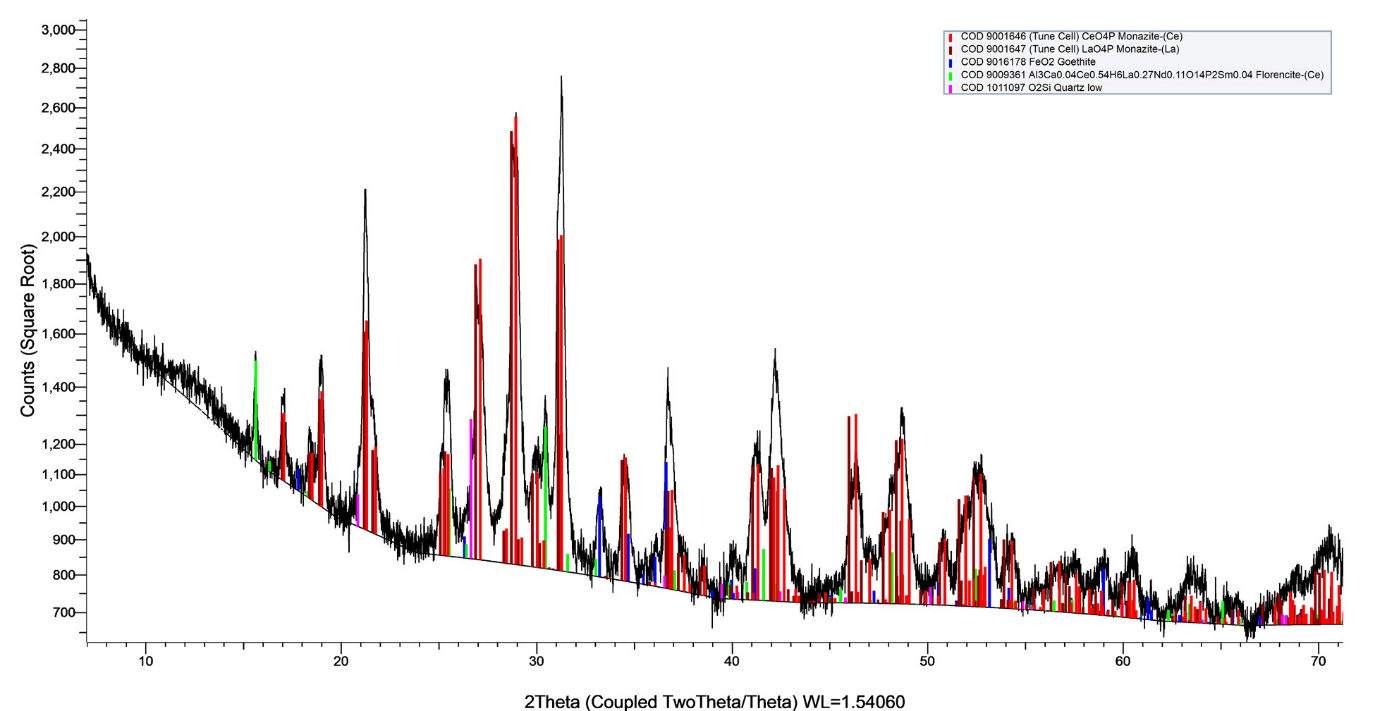


**Figure S-2.** XRD phase identification of the high-grade monazite ore conducted at John de Laeter Centre , Curtin University.

**Table S-2.** XRD and phase identification of the high-grade monazite ore conducted by John de Laeter Centre, Curtin University. The COD ID refers to the phase’s identification number in the COD database (<http://www.crystallography.net/>)

| **Phase** | COD ID | Nominal elemental composition |
| --- | --- | --- |
| **Monazite, Ce** | 9001646 | CePO_4_ |
| **Monazite, La** | 9001647 | LaPO_4_ |
| **Quartz** | 1011097 | SiO_2_ |
| **Goethite** | 9016178 | FeOOH |
| **Florencite-Ce** | 9009361 | Al_3_(Ce,La,Nd,Sm,Ca)(PO_4_)_2_(OH)_6_ |

**Note**: Since crandallite group CaAl₃(PO₄)(PO₃OH)(OH)₆ and florencite peaks may overlaps, another QXRD analyses was done by Bureau Veritas (Perth, WA).

**Table S-3.** Quantitative XRD results (Crystalline phases only - wt%) of high-grade monazite. conducted by Bureau Veritas, Perth, Australia.

| **Mineral** | **Monazite Group** | **Kihlmanite** | **Crandallite group** | **Alunite group** | **Total** |
| --- | --- | --- | --- | --- | --- |
|  | 65 | 3 | 15 | 18 | 100 |

**Table S-4.** The inductively coupled plasma mass spectrometry, and TIMA composition analysis of high-grade monazite ore conducted by Bureau Veritas, Perth, Australia.

|  | **Elements (%)** |  |
| --- | --- | --- |
| **Al** | | 3.9 |
| **Ca** | | 1.8 |
| **Fe** | | 1 |
| **K** | | <0.01 |
| **Mg** | | 0.1 |
| **Mn** | | 0.06 |
| **Na** | | 0.1 |
| **P** | | 8.5 |
| **Si** | | 1.6 |
| **Ti** | | 0.4 |
| **Y** | | 0.18 |
| **La** | | 11 |
| **Ce** | | 15 |
| **Pr** | | 2.1 |
| **Nd** | | 7.2 |
| **Sm** | | 0.97 |
| **S** | | 0 |


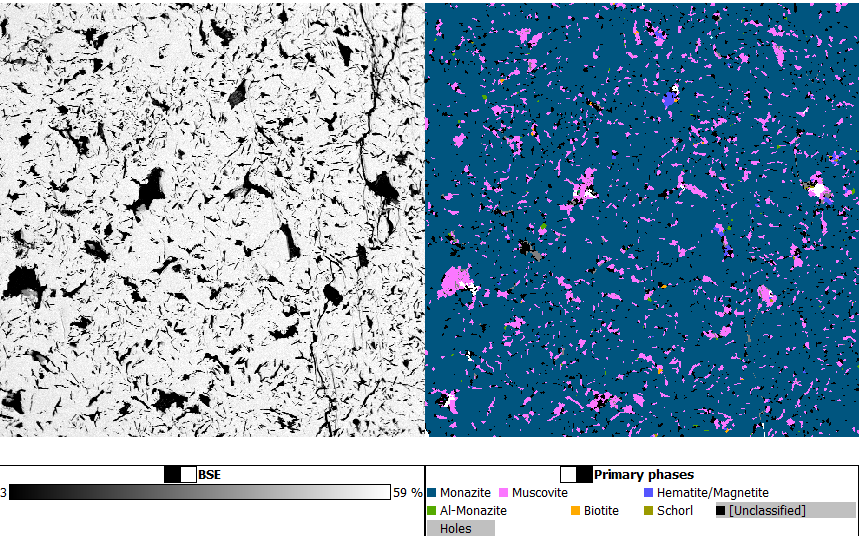


**Figure S-3.** Quantitative TIMA mineral mapping of monazite-muscovite crystals.


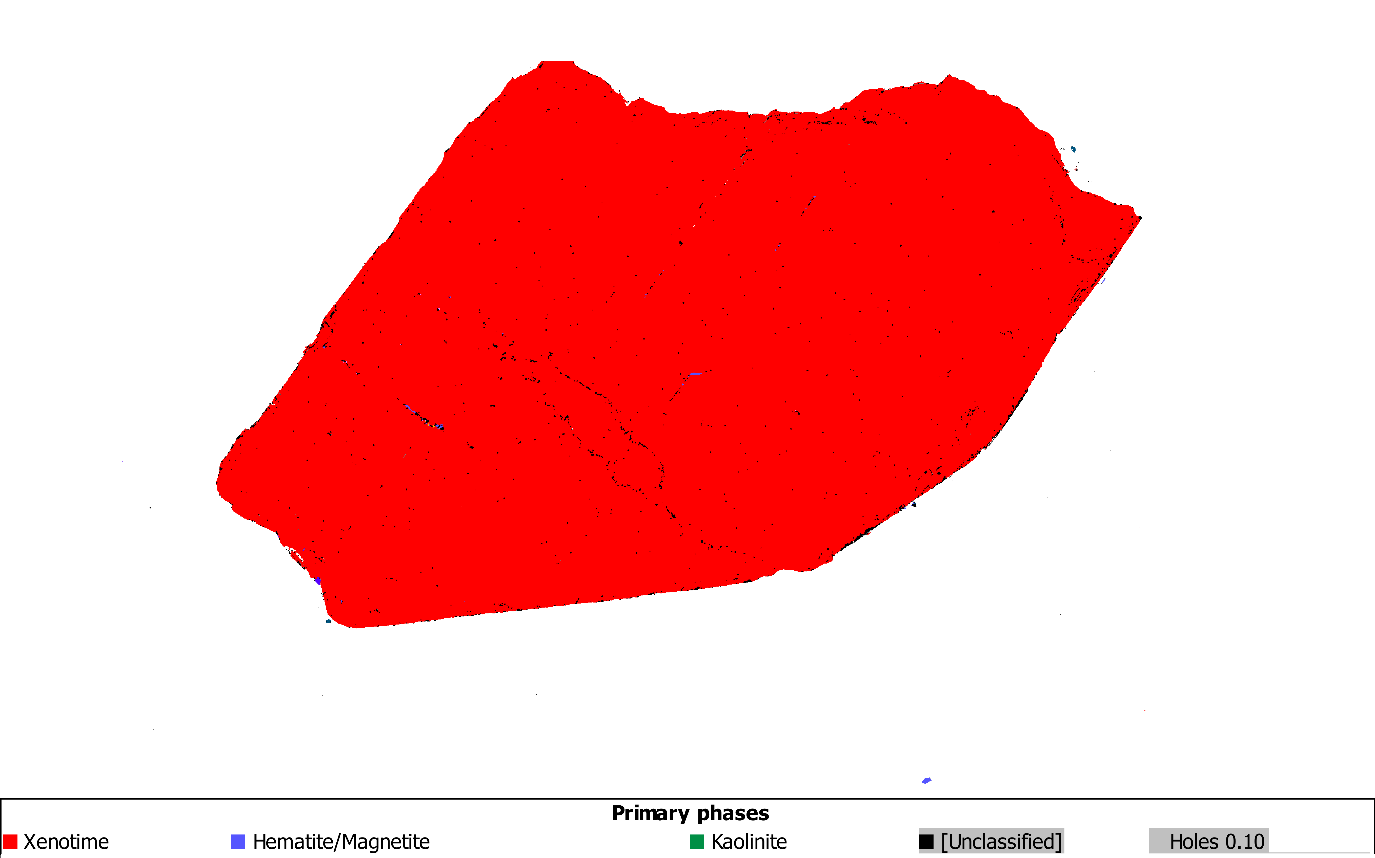


**Figure S-4.** TIMA mineral mapping of xenotime crystals.

**
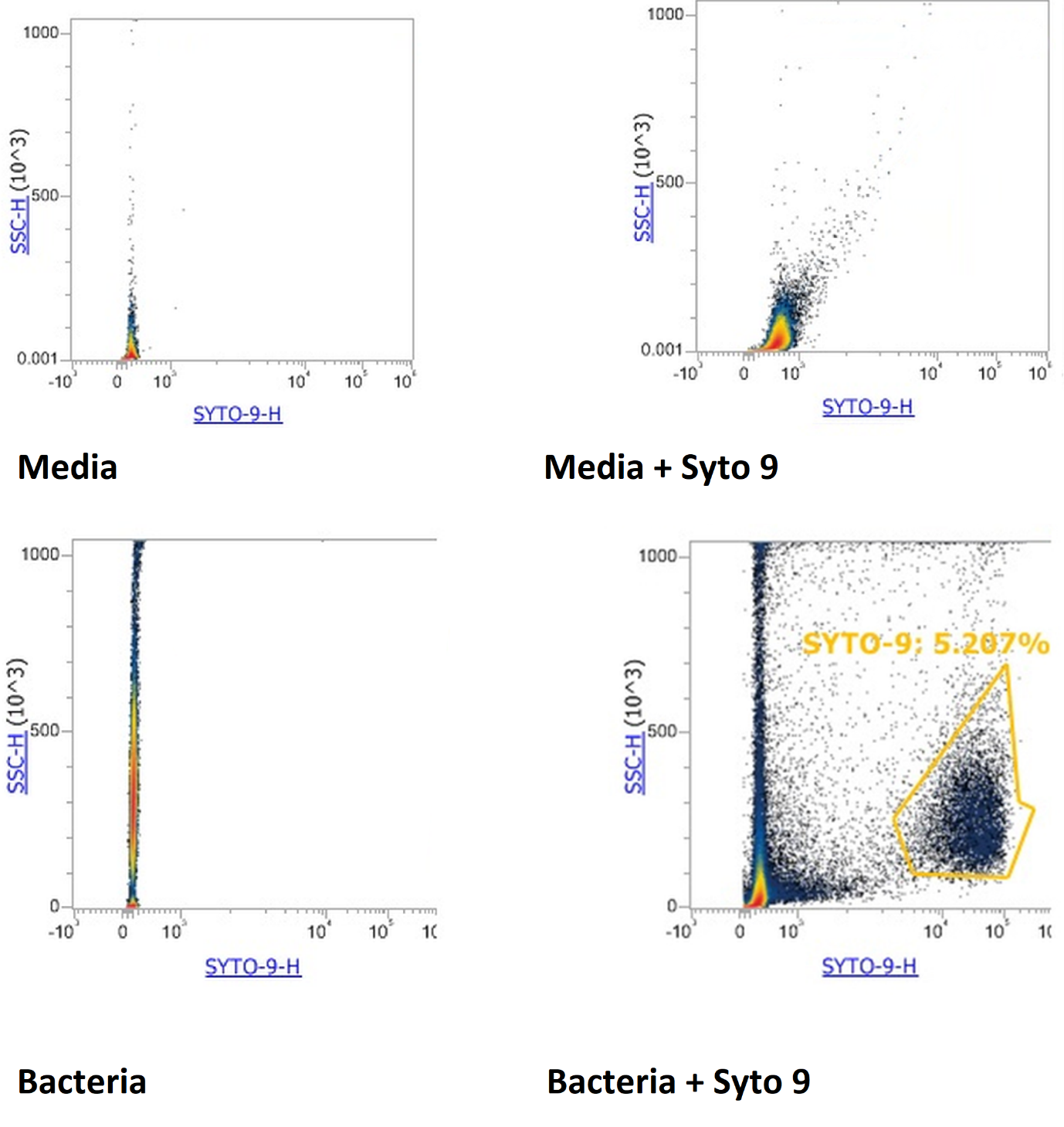
**

**Figure S-5.** The flow cytometry plots for sterile media and bacteria with and without SYTO9 as the fluorochrome for DNA.


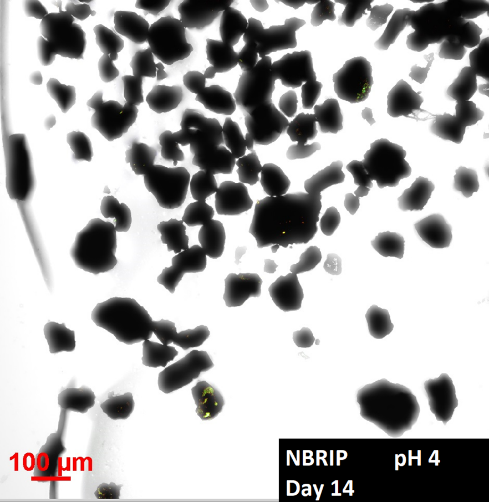

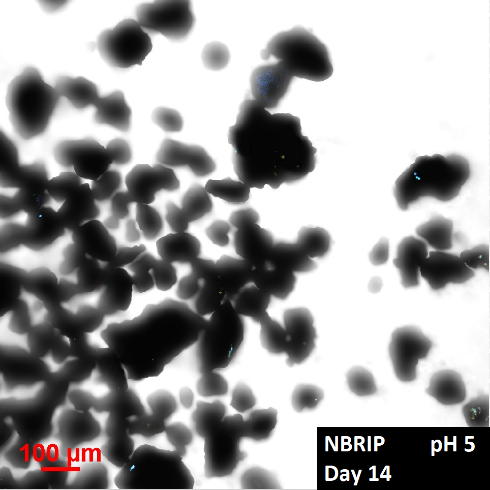

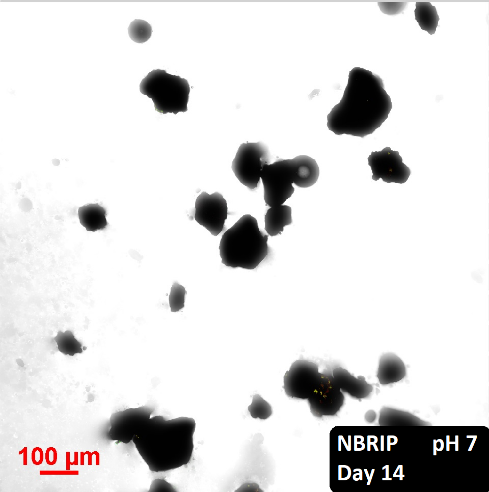


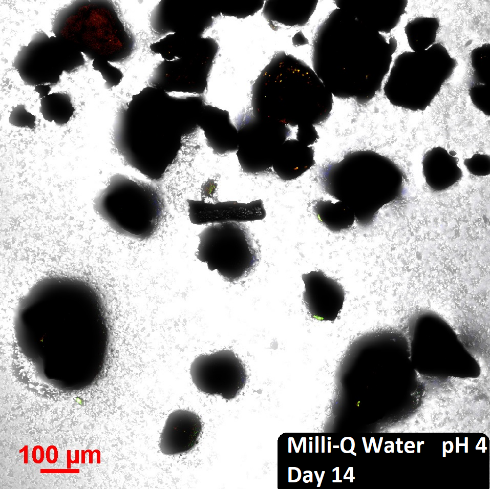

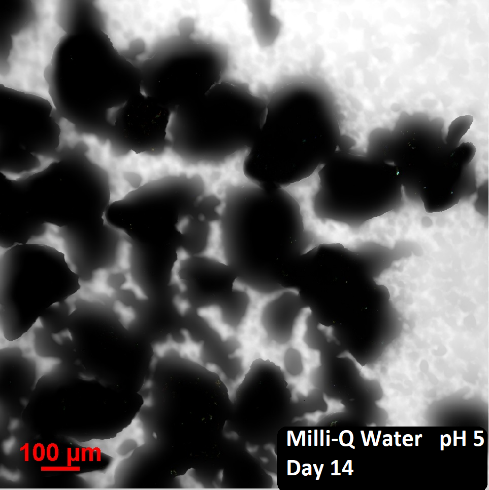

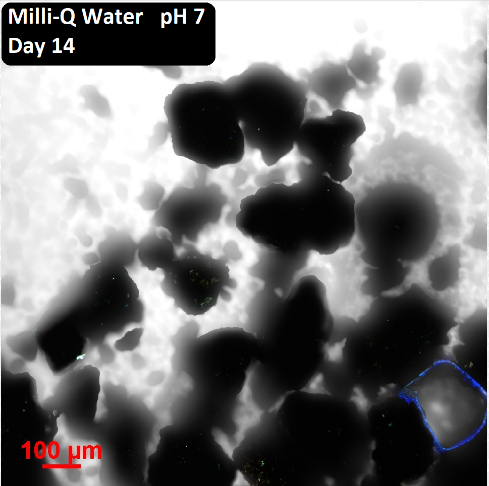


**Figure S-6.** The recorded autofluorescence of high grade monazite ore after 14 days of abiotic leaching using NBRIP and Milli-Q water at pH 4, 5 and 7.


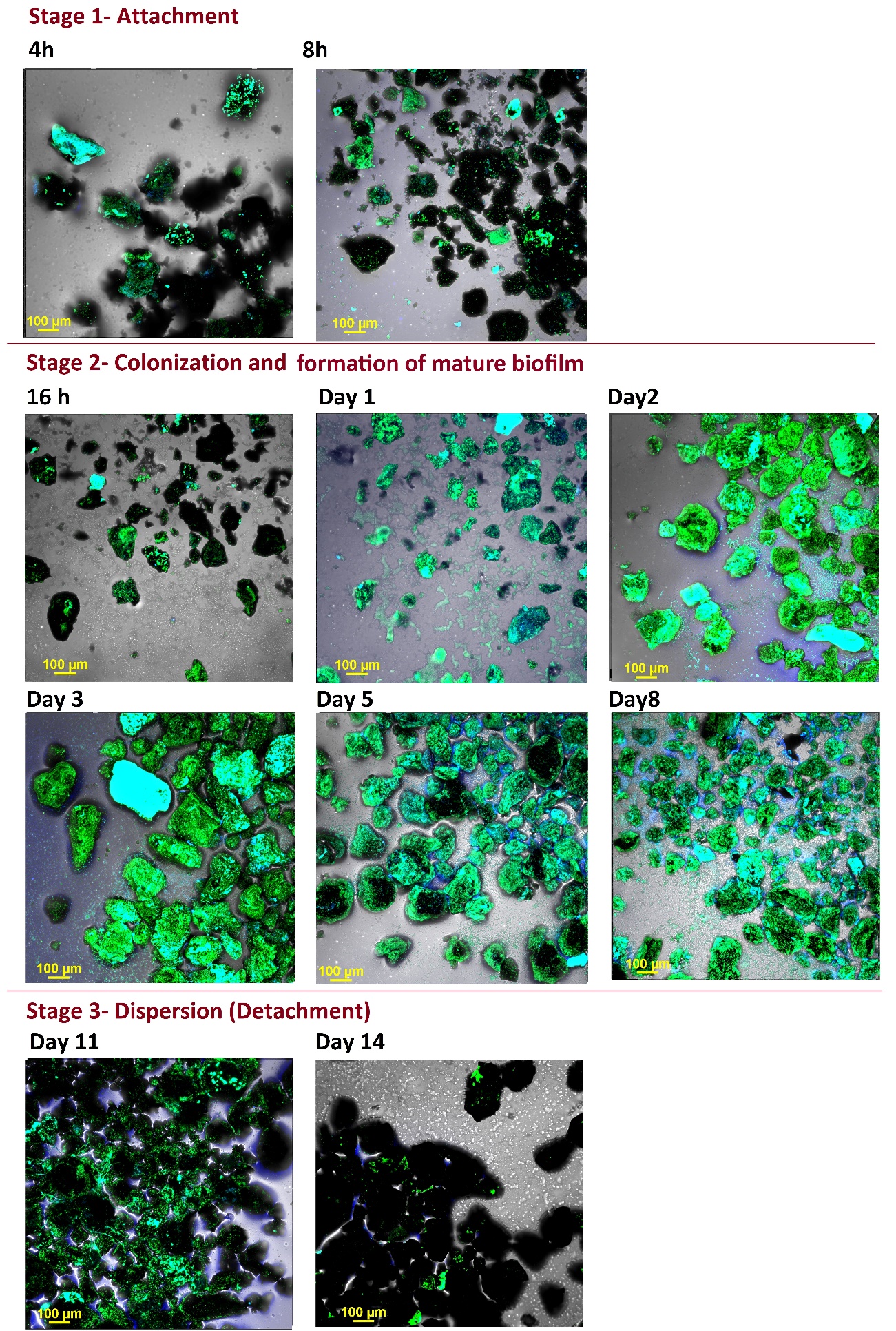


**Figure S-7.** Visualisation of *Klebsiella aerogenes* biofilm on the surface of high grade monazite ore by CLSM. Hoechst 33342 (blue) and DiTO-1 (green) were used to maximum visualisation of the bacterial cells. CLSM image is a merged image (blue, green and transmitted light channels) using the Maximum Fluorescent Intensity of the acquired Z-stack images. Blue, green or cyan colours on the surface of the mineral grains (the dark grains) are representative of the microbial biofilm. The background blue colour around the mineral grains is autofluorescent from the epoxy glue.


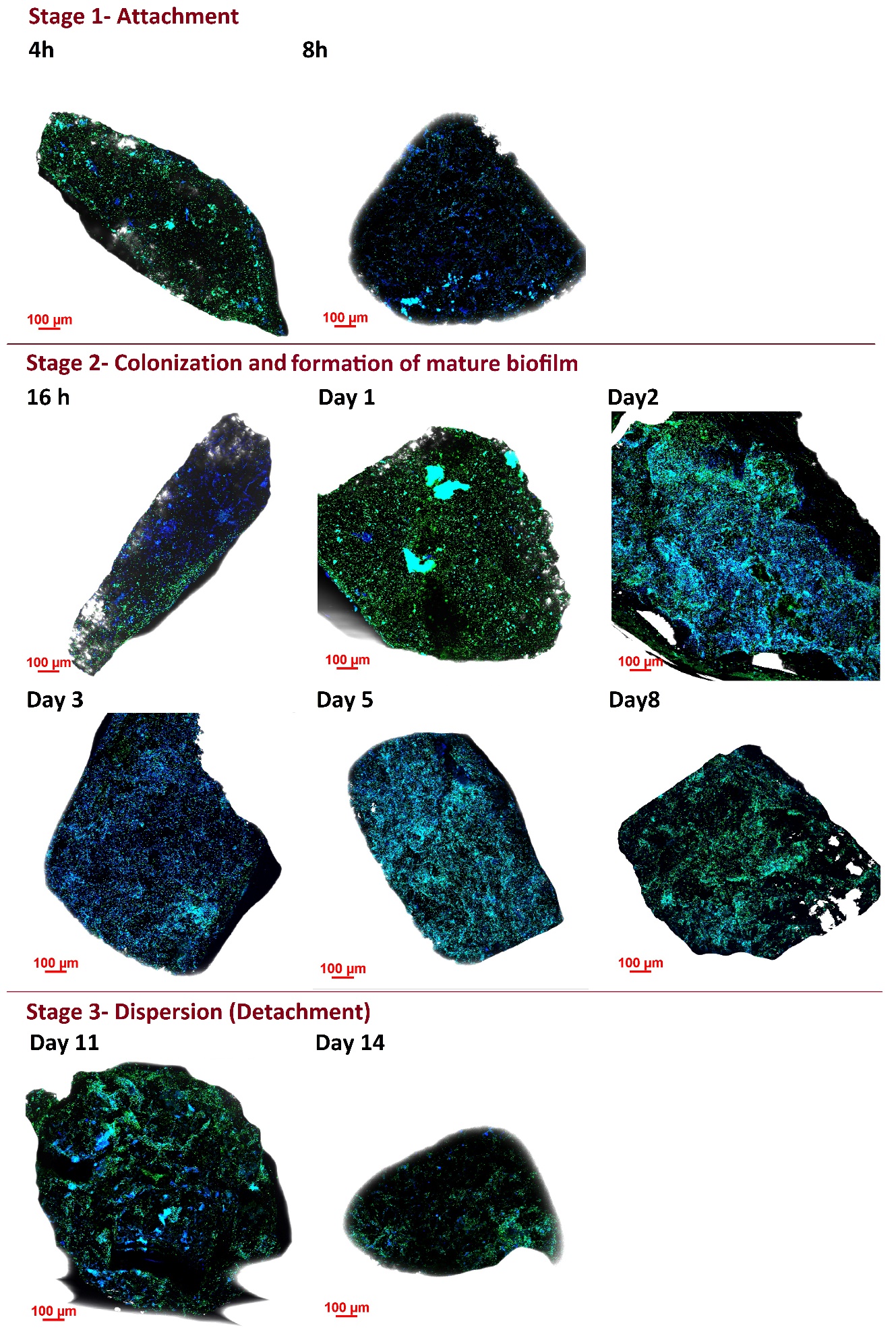


**Figure S-8.** Visualisation of *Klebsiella aerogenes* biofilm on the surface of monazite-muscovite crystals by CLSM.


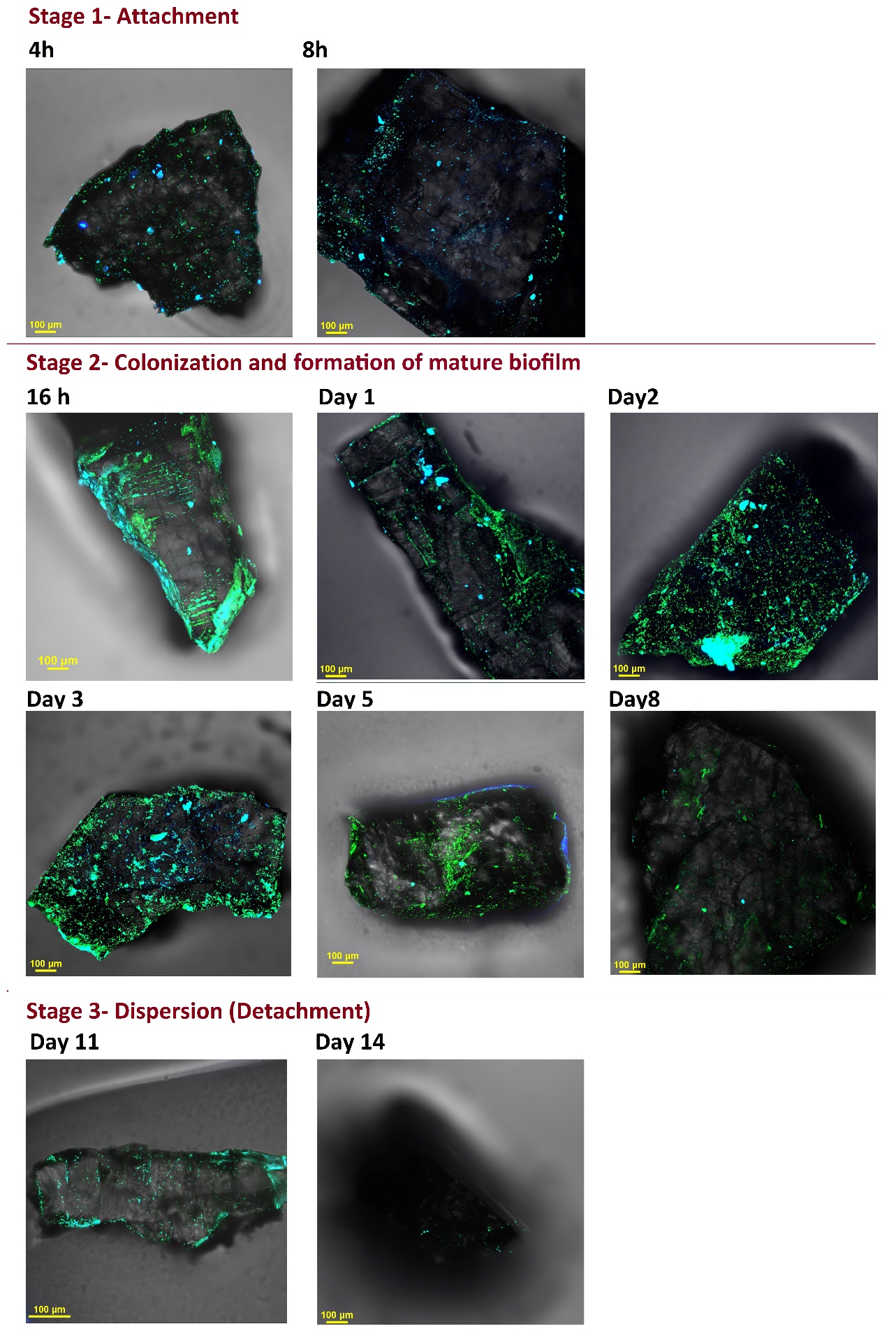


**Figure S-9.** Visualisation of *Klebsiella aerogenes* biofilm on the surface of xenotime crystals by CLSM.

**
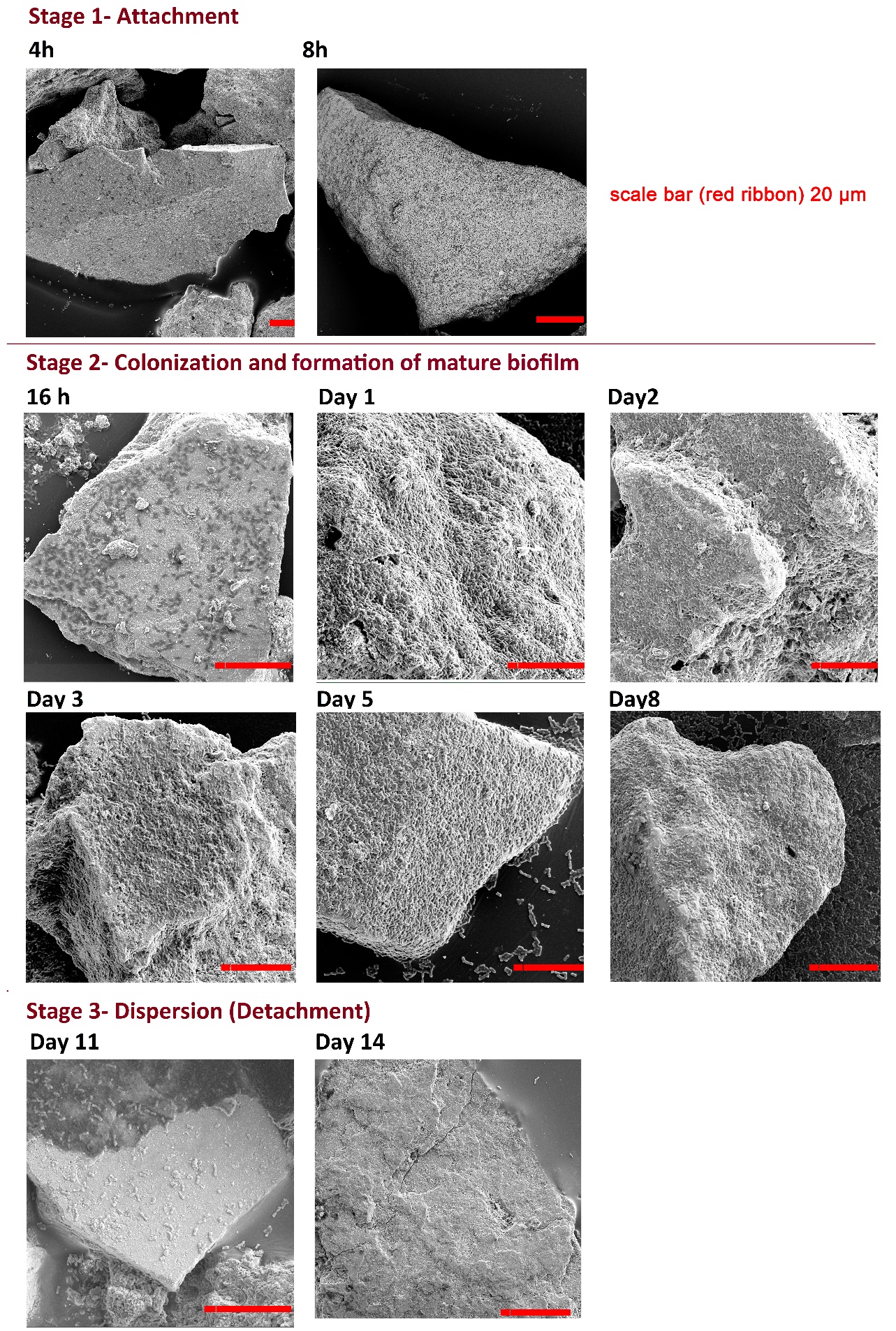
**

**Figure S-10.** Visualisation of *Klebsiella aerogenes* biofilm on the surface of high grade monazite ore by SEM.


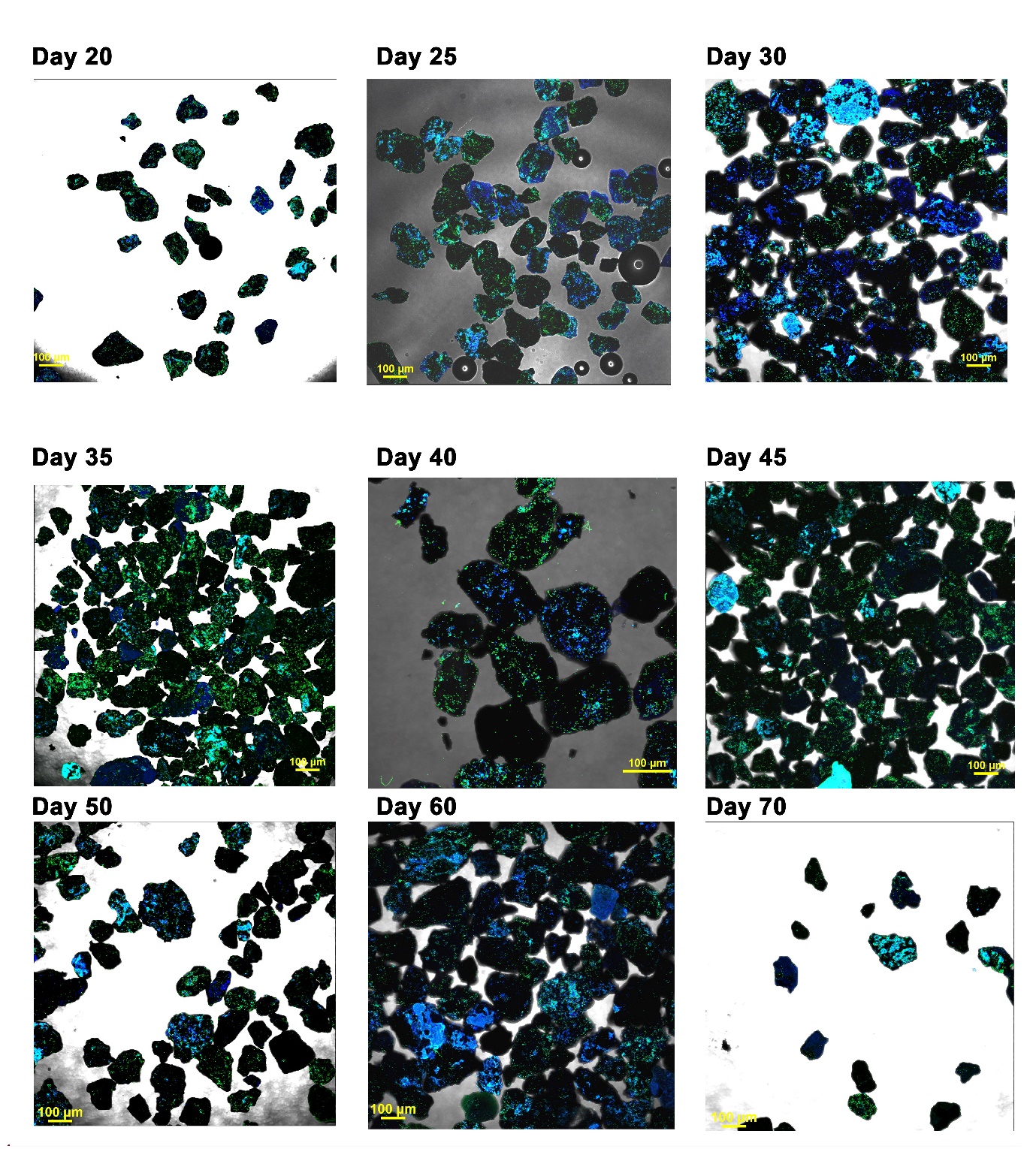


**Figure S-11.** Visualisation of *Klebsiella aerogenes* biofilm on the surface of high grade monazite ore, day 20-70.

**
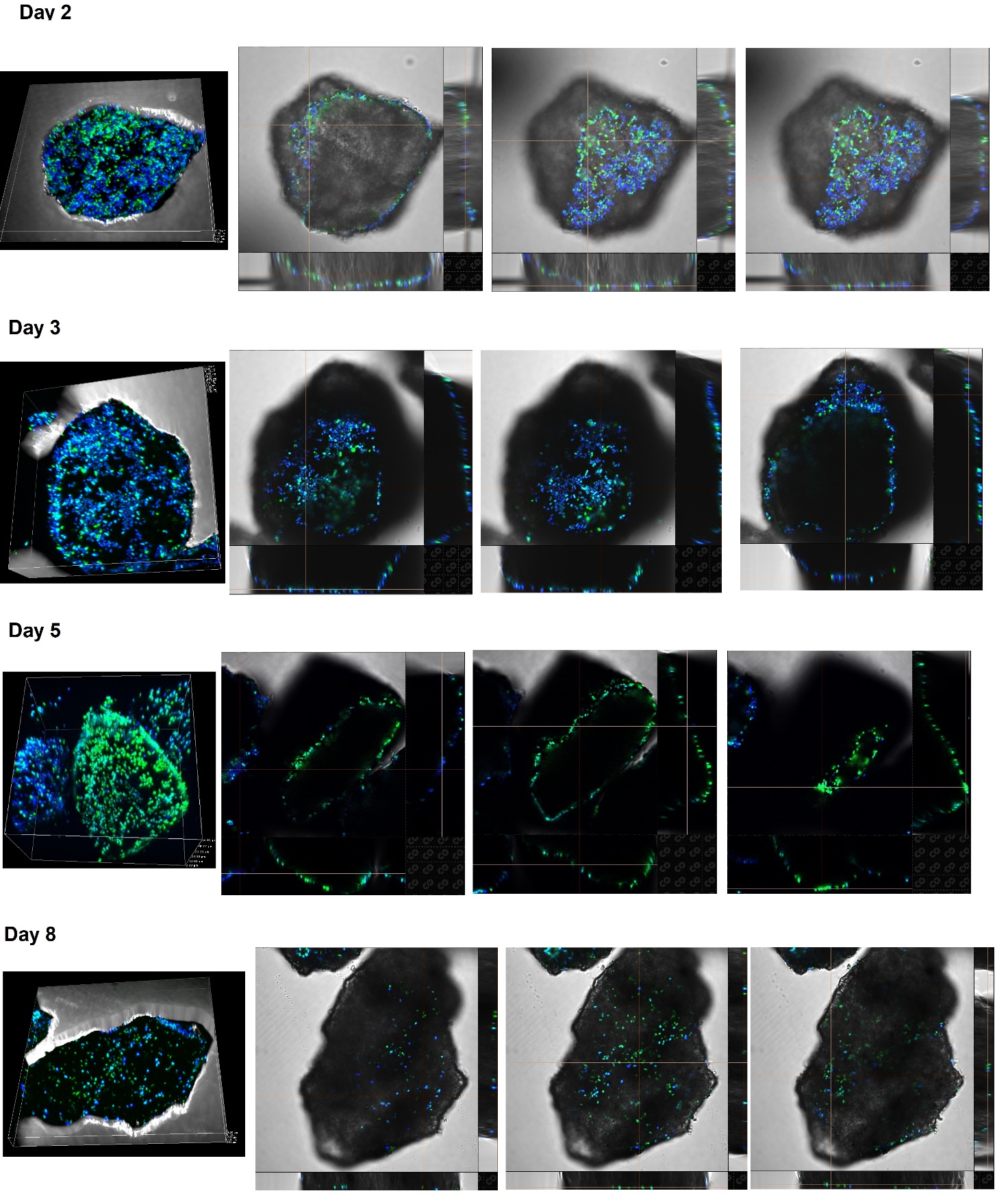
**

**Figure S-12.** Figure S-12. CLSM cross-section of *K. aerogenes* mature biofilms on high grade monazite ore. The blue/green shapes in the CLSM images are representative of the microbial cells.


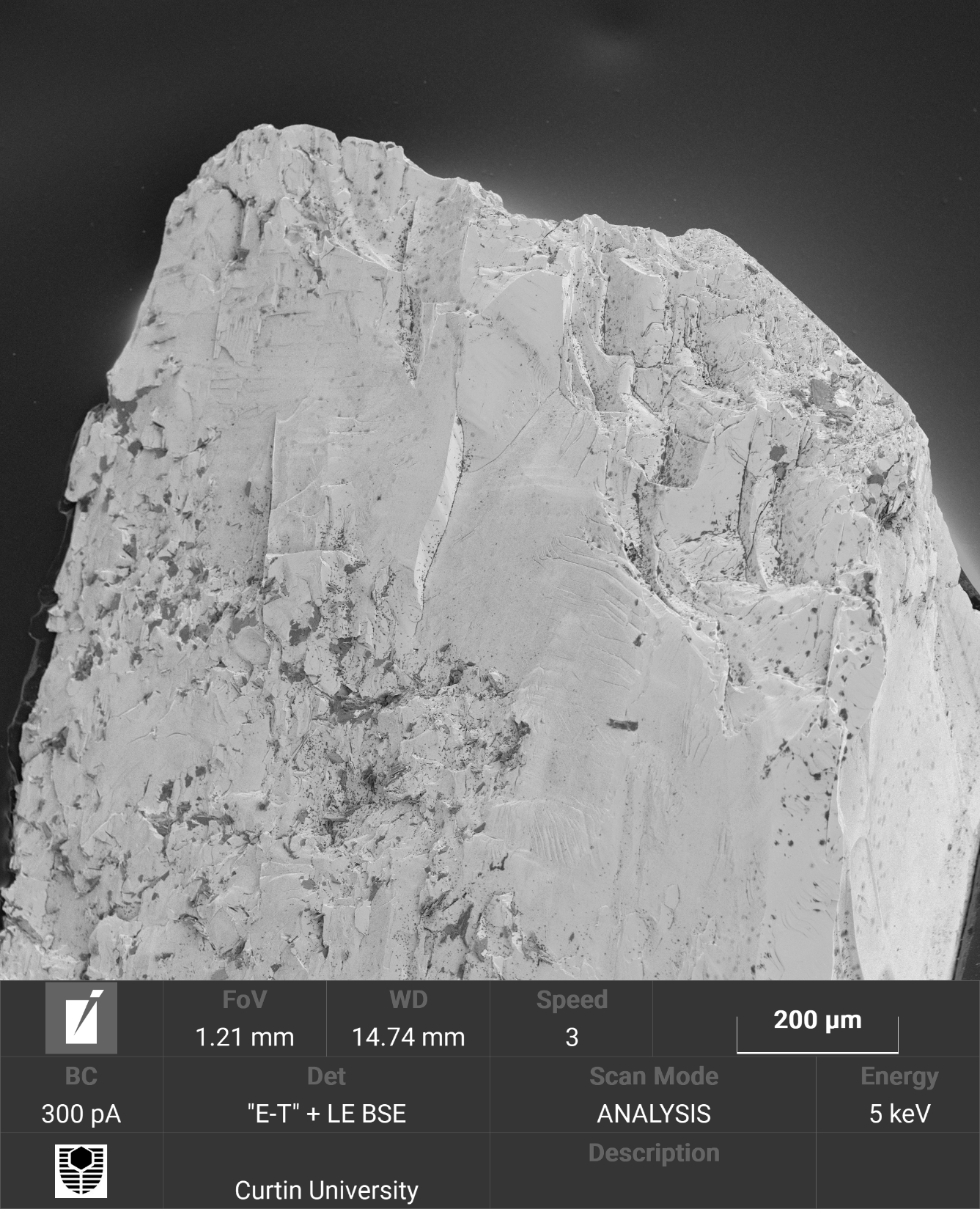


200 µm

a

100 µm

b


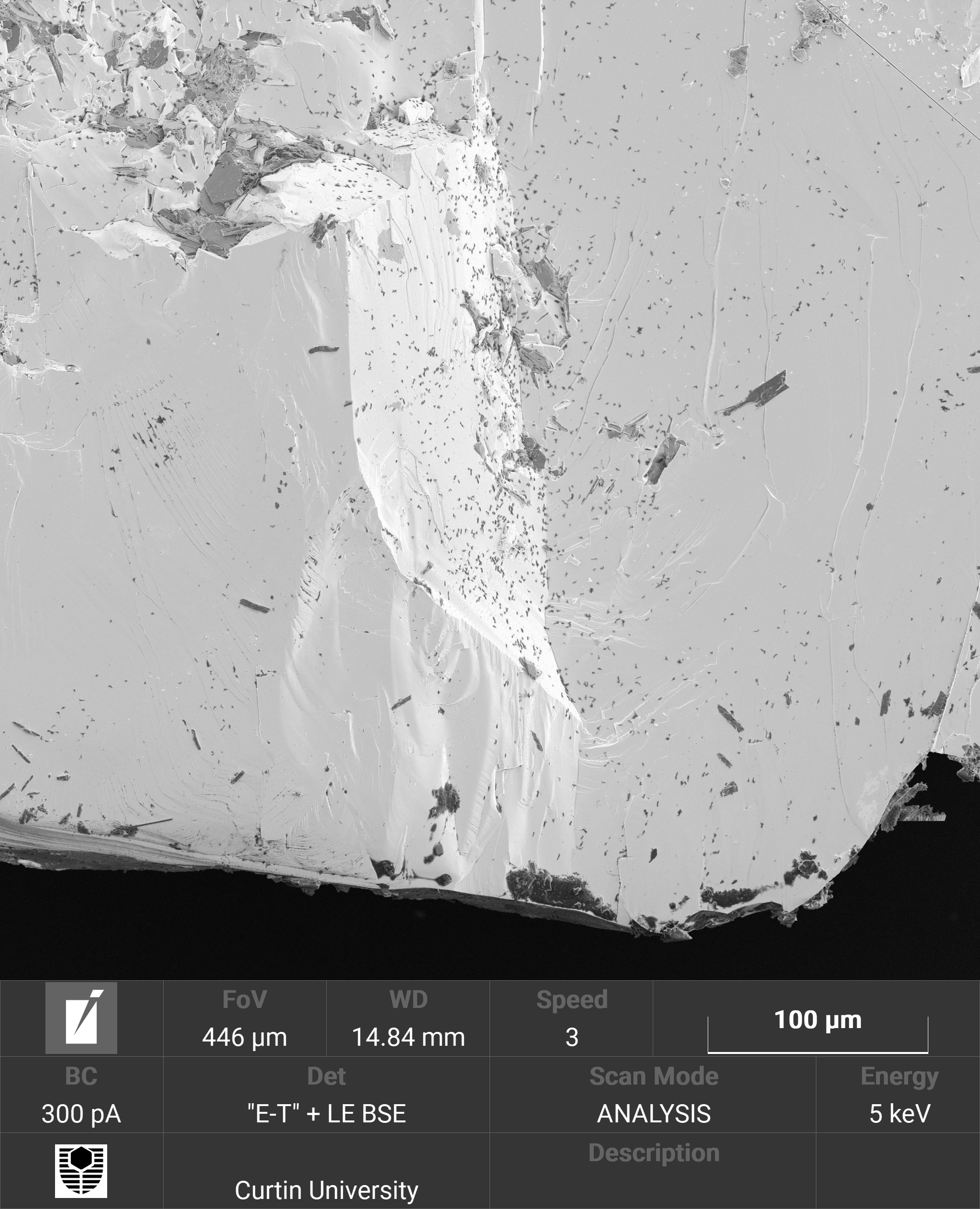


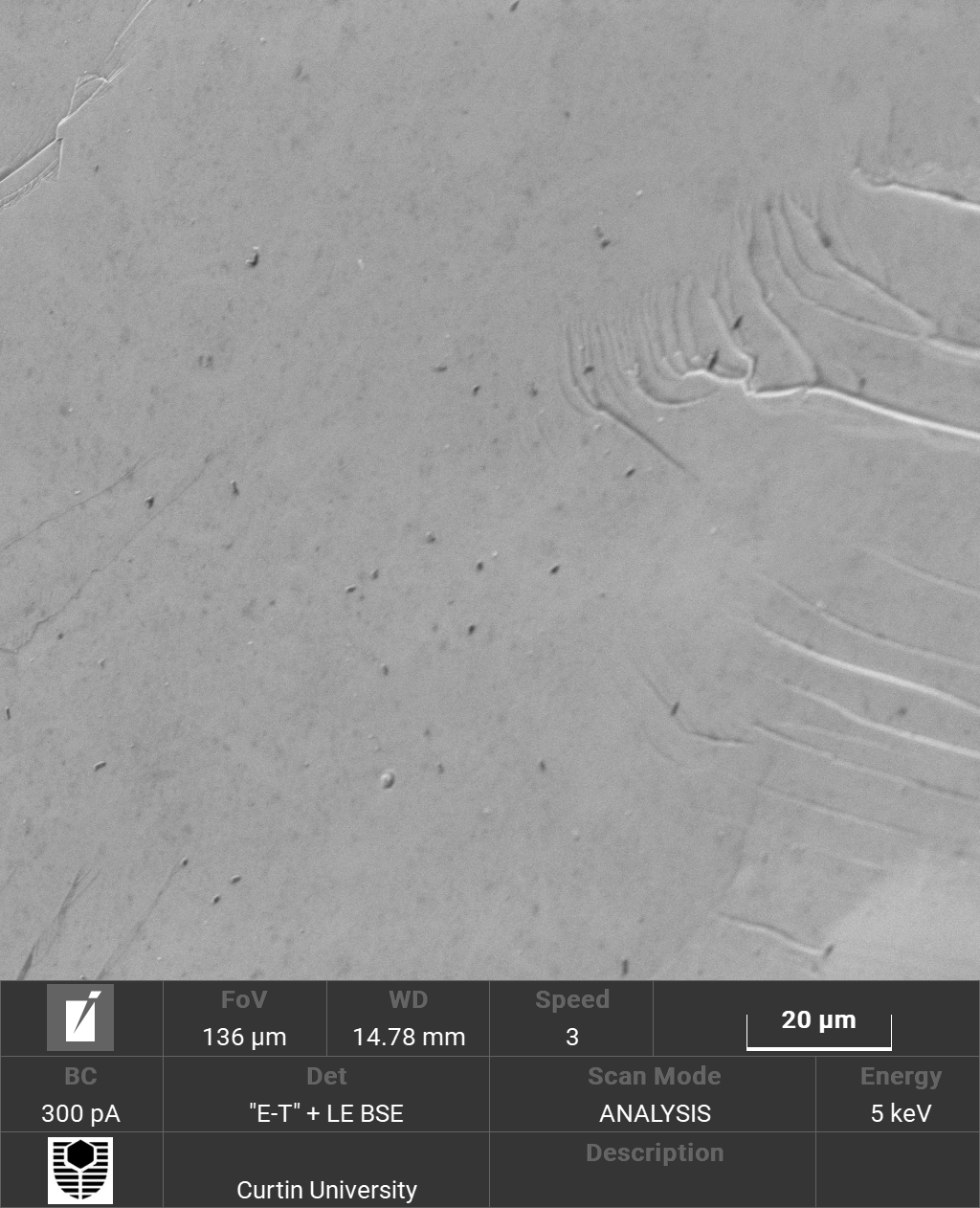


20 µm

c


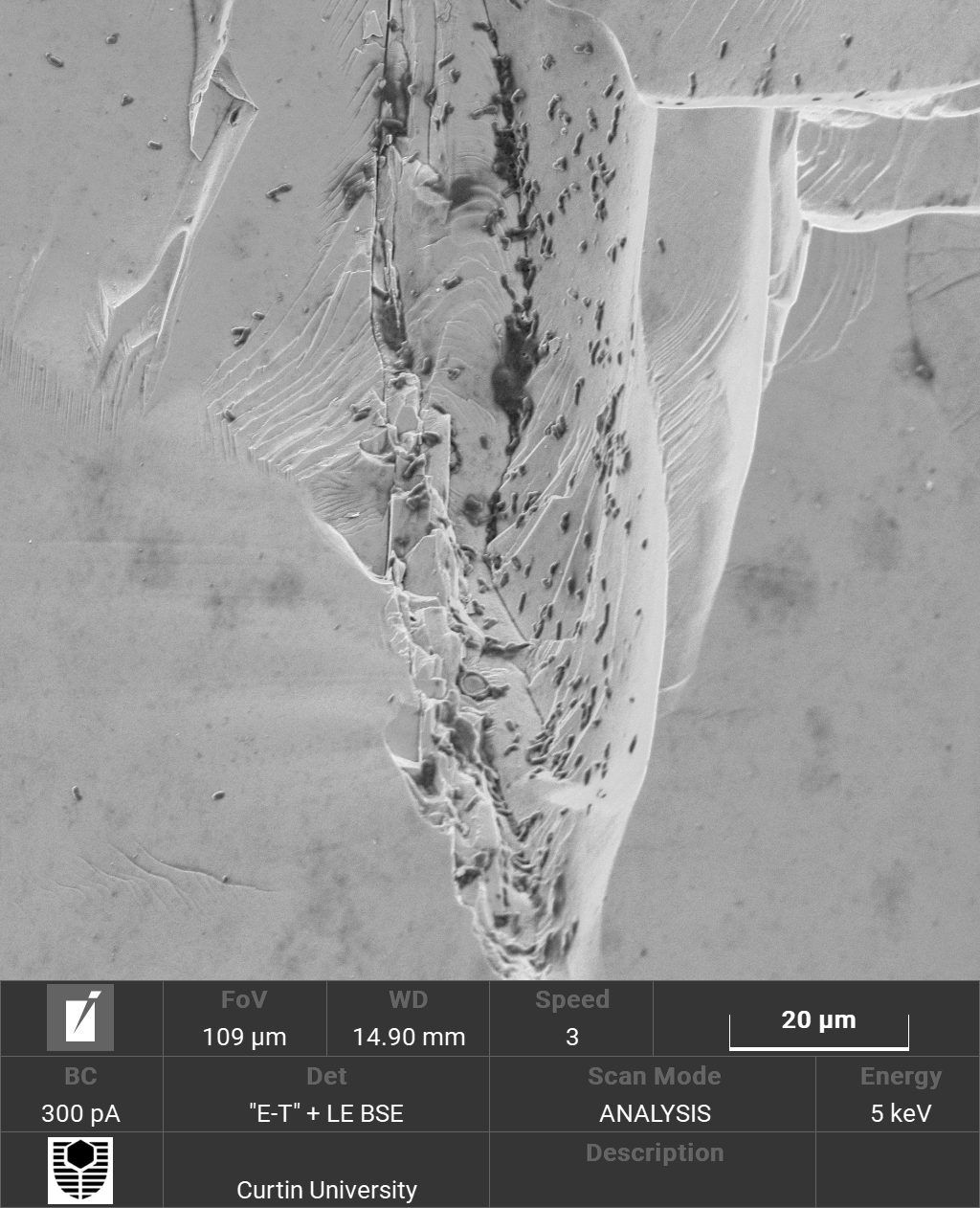


20 µm

d

20 µm

e


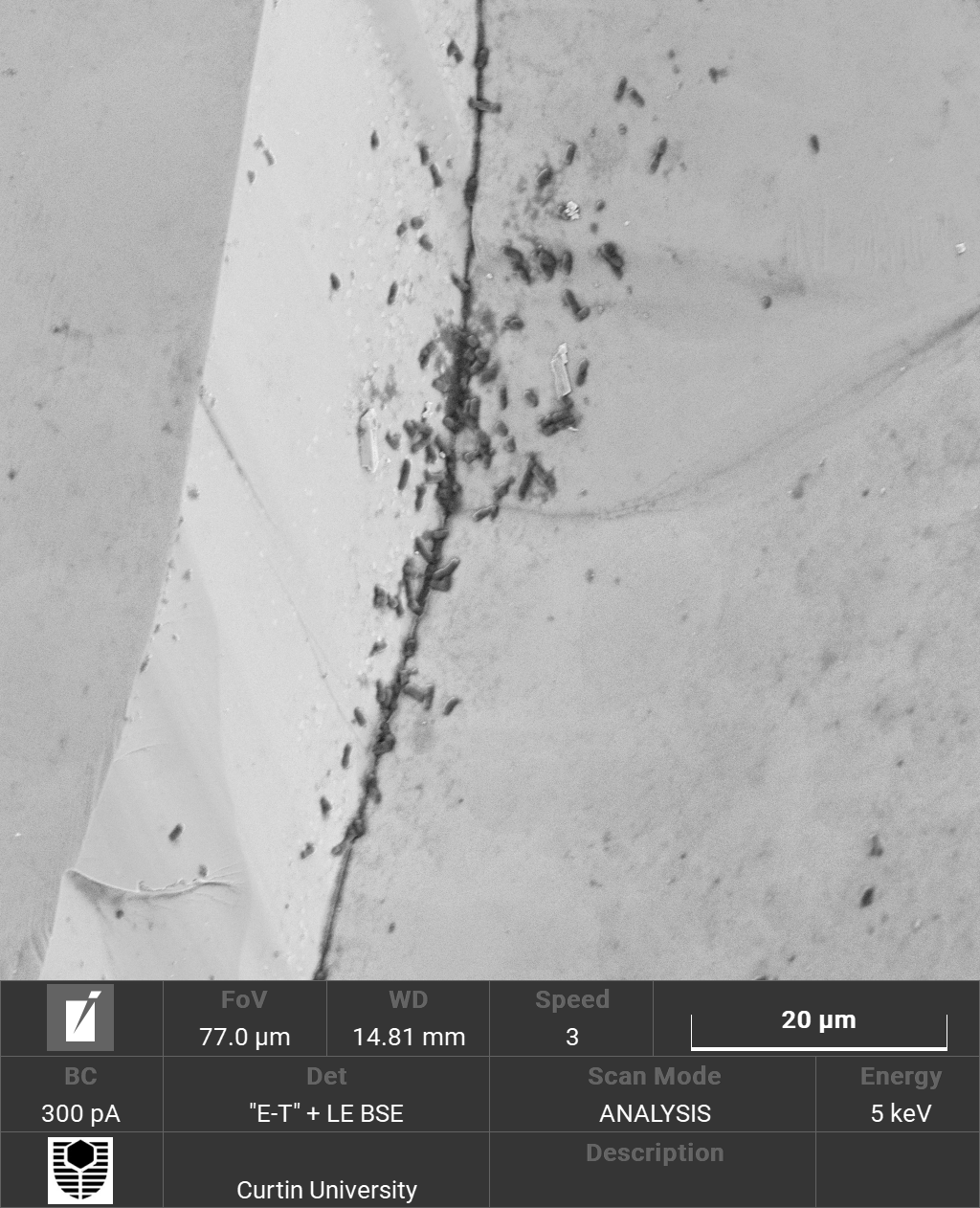


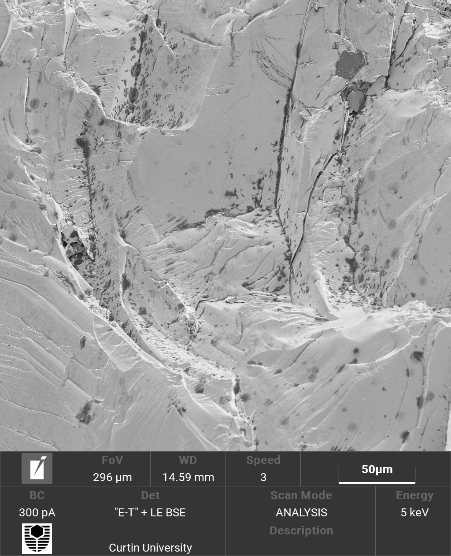


50 µm

f


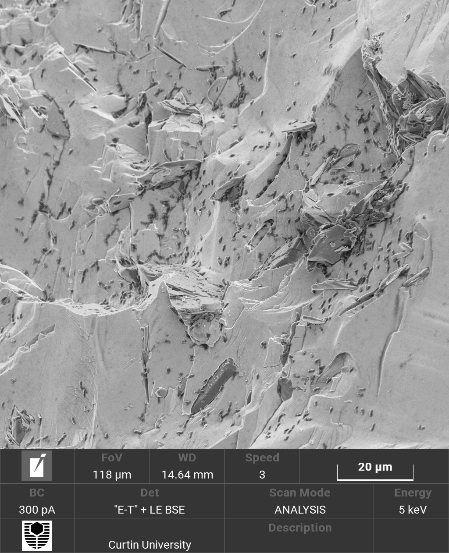


20 µm

g

**Figure S-13.** Localization of *K. aerogenes* biofilm on and around the physical surface imperfections of monazite-muscovite crystal. (a) An overall SEM of the crystal. (b) A location on the crystal with an imperfection site (ridge) in the centre and fairly flat surfaces on the left and right of the ridge. (c) A location on the crystal with flat surface. (d-g) Other imperfection sites on the surface.


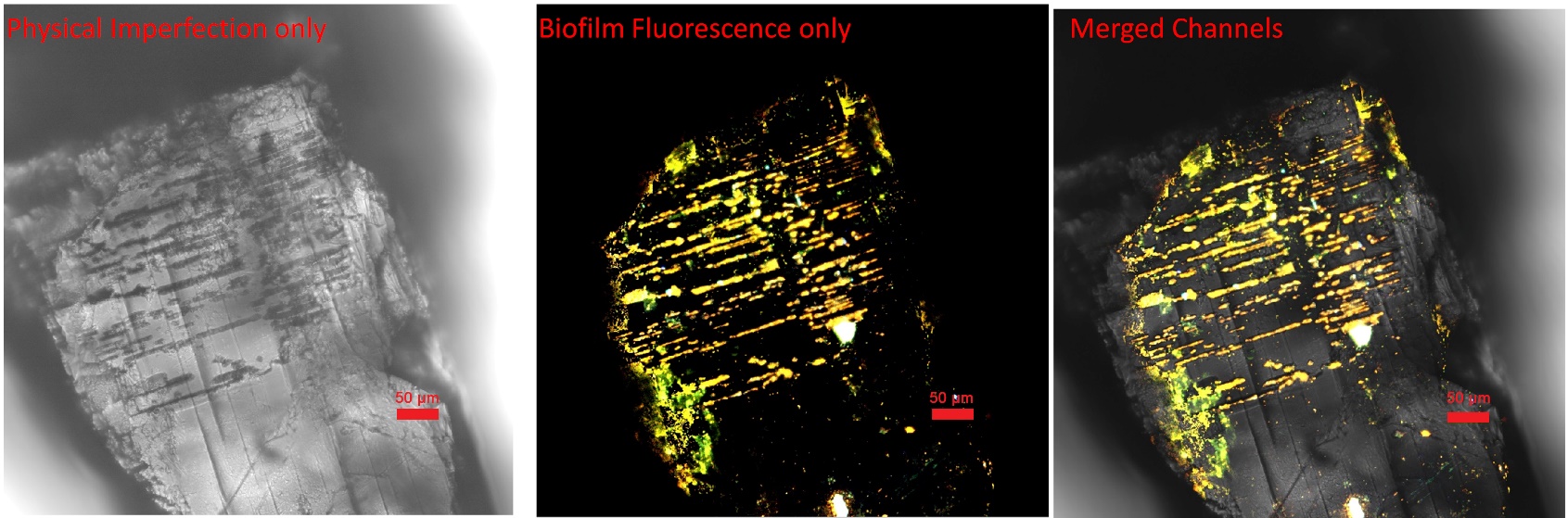


a


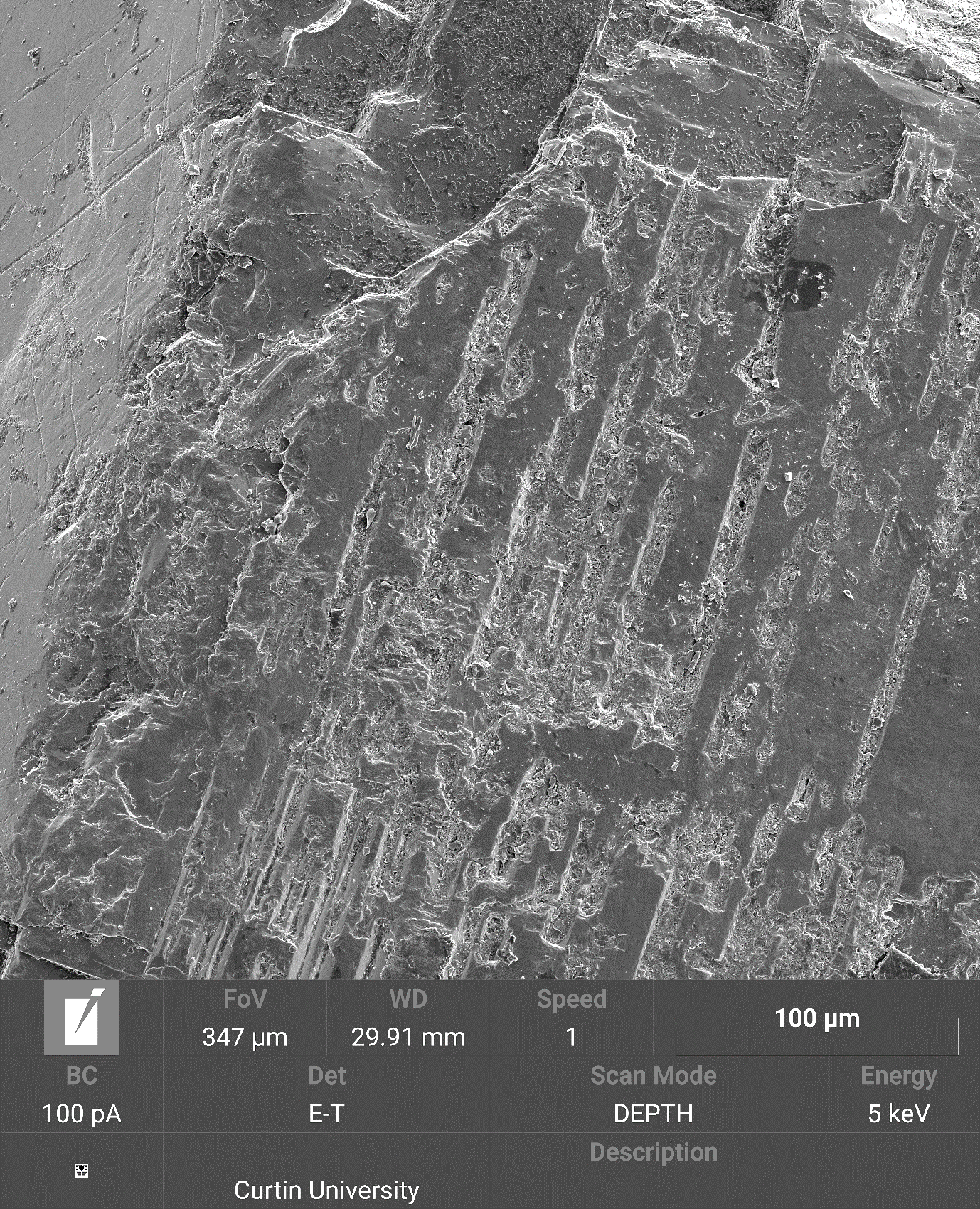


100 µm

b


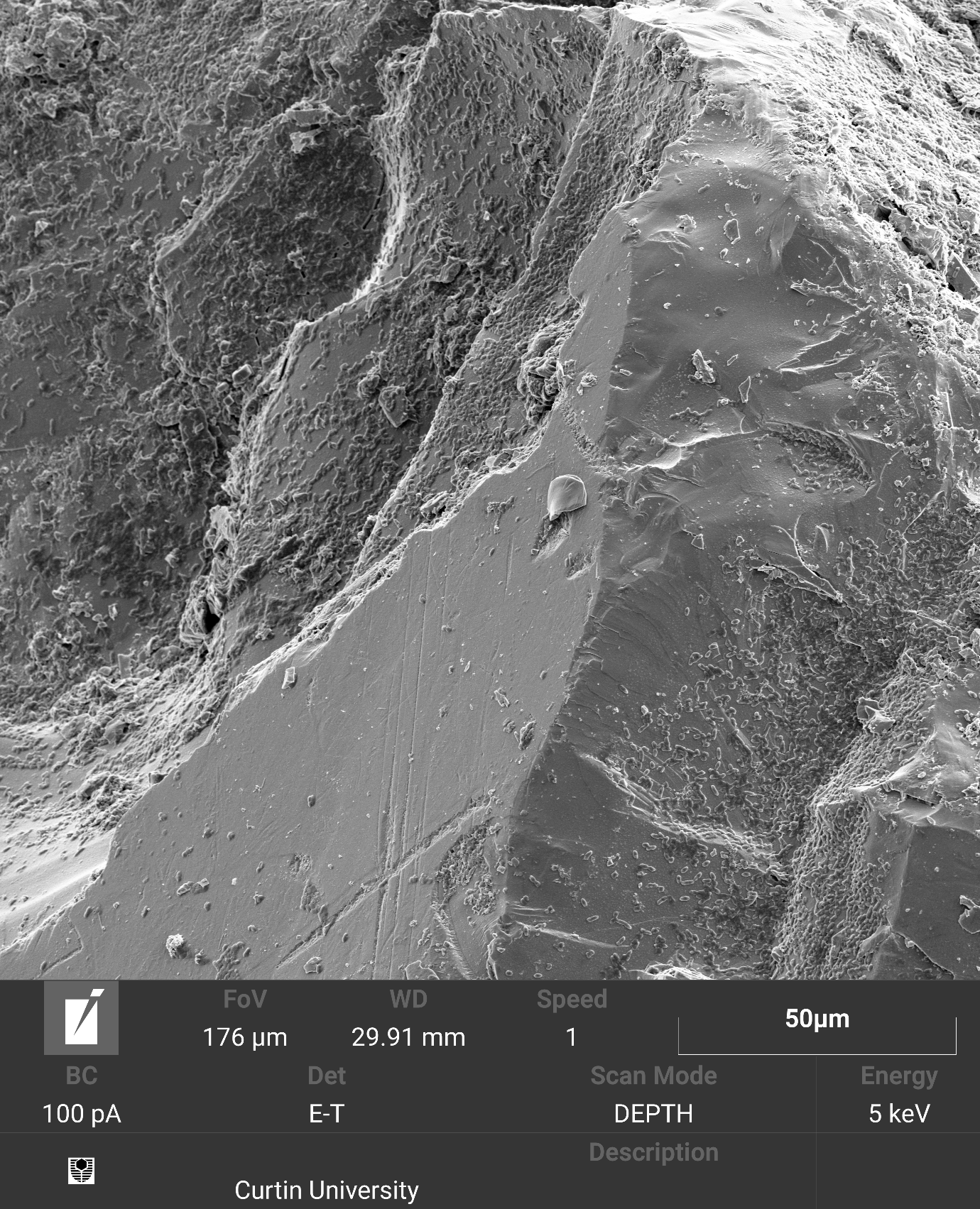


50 µm

c


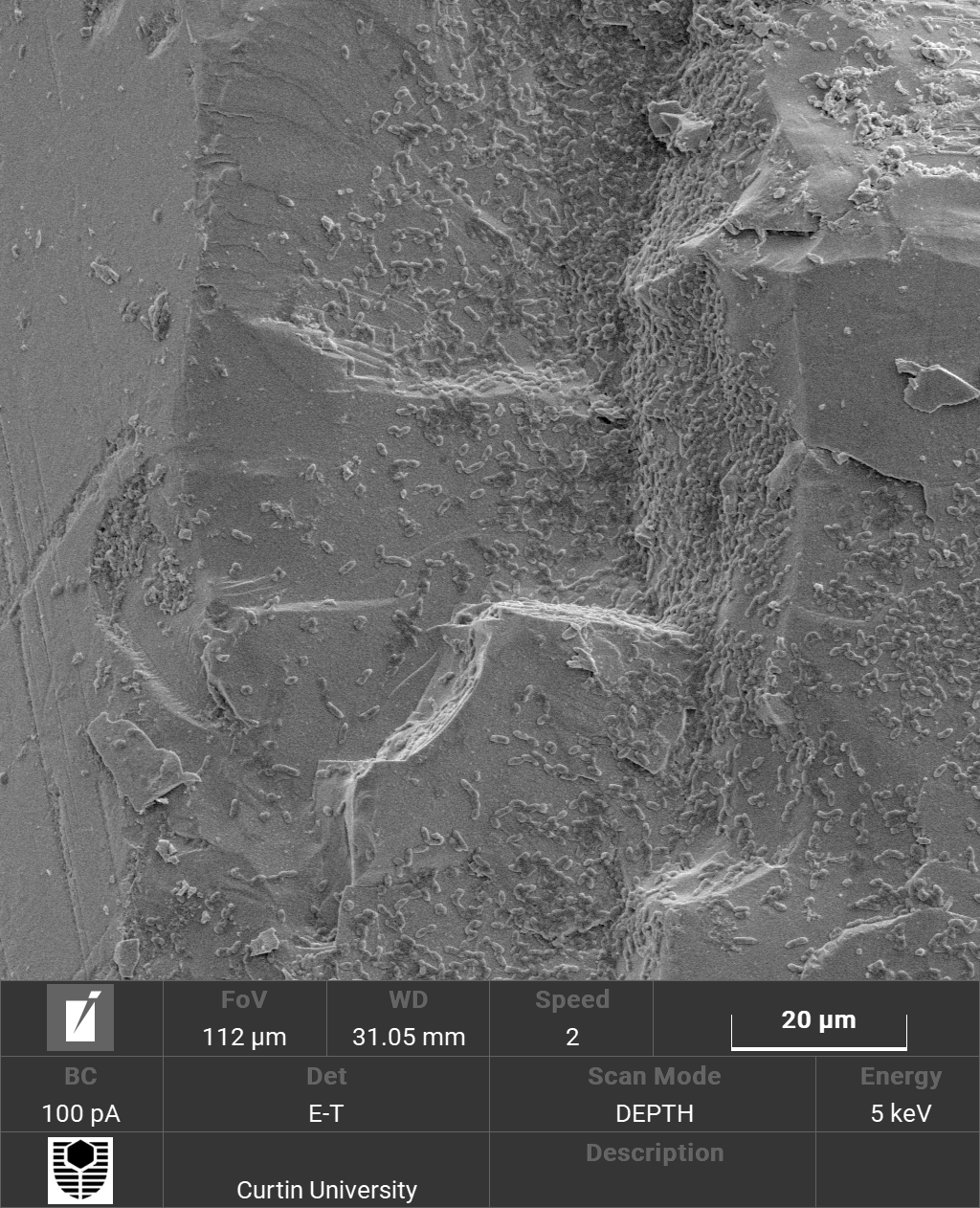


20 µm

d


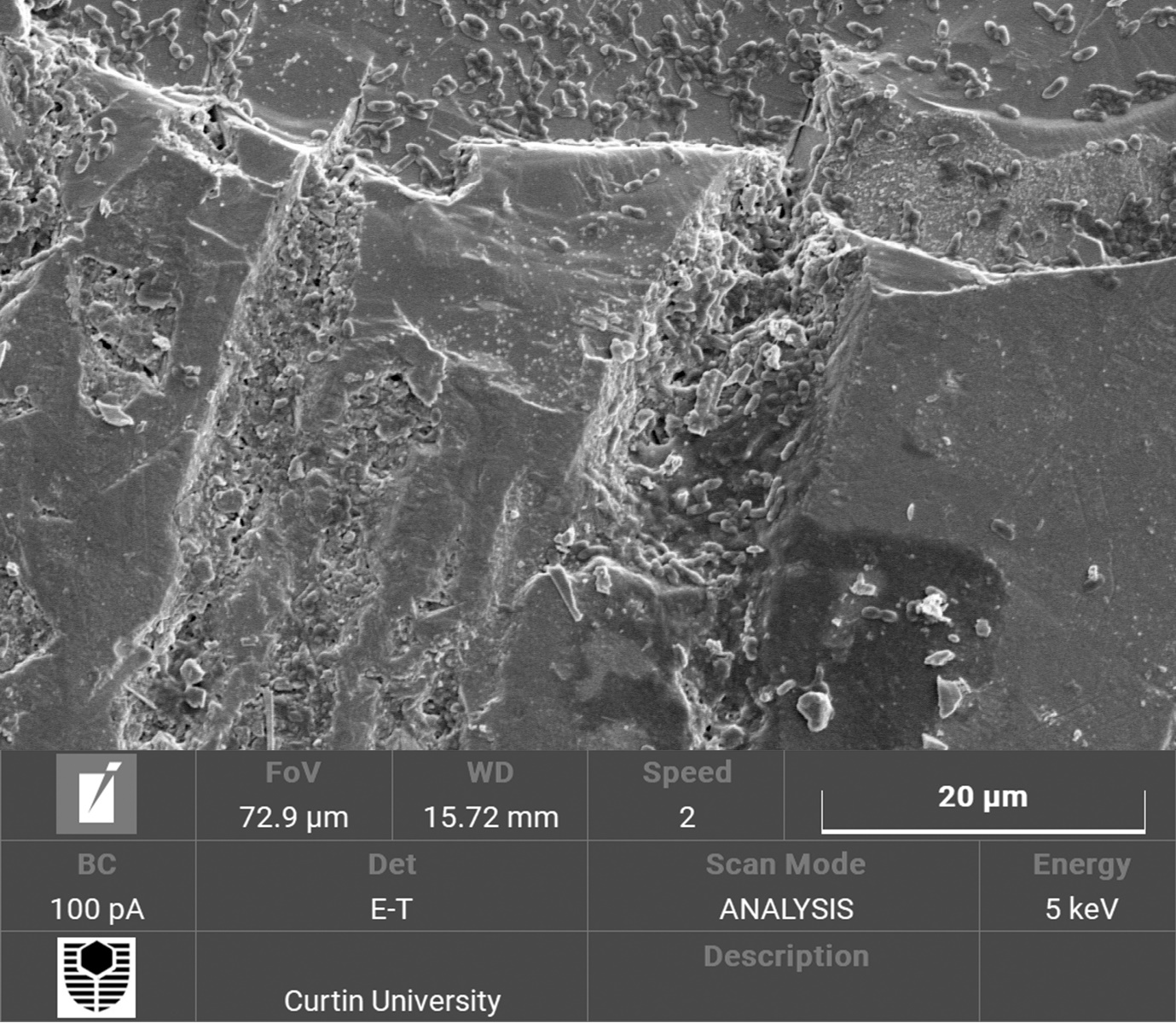


20 µm

e

**Figure S-14.** Localization of *K. aerogenes* biofilm around the physical surface imperfections of xenotime crystal. (a) An overall CLSM image of the crystal surface in transmission light mode (physical imperfection only), florescence channel only, and merged image. (b-e) The SEM images of the same sample zooming on a smaller area (red box in panel-a) at different magnification.

**Video S-1.** *Klebsiella aerogenes* biofilm on the surface of high grade monazite ore monazite acquired using Z-stack imaging at maximum fluorescent intensity. The acquired Z-stack images are shown in a depth-coded rainbow coloured 3D image. The depth rainbow coloured area is representative of the biofilm and bacterial cells on the surface of the mineral. The colouring scheme represents the height of the sample where blue is closer to the top of the mineral grain and pink represent higher depth and is closer to the bottom of the mineral.


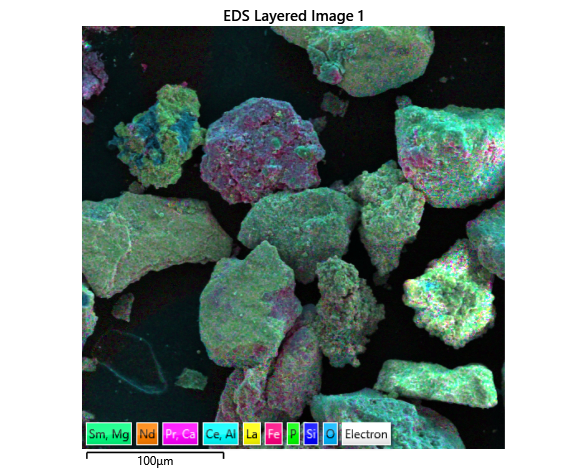


a


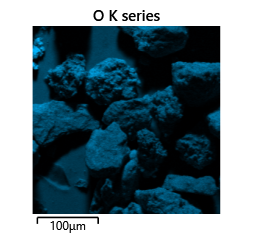

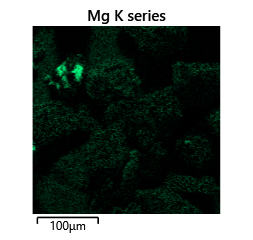


b

c


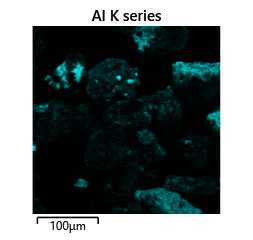

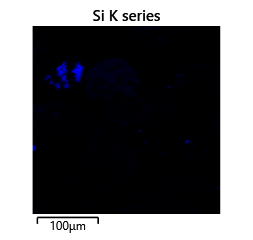


d

e


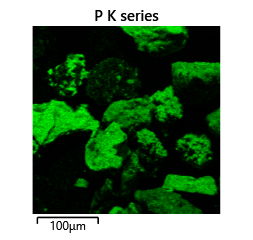

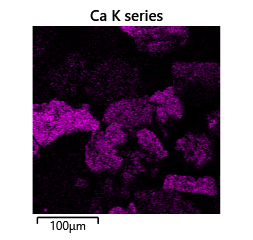


f

g


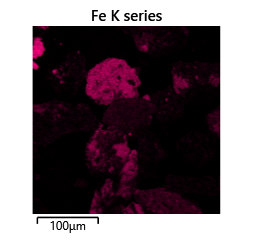

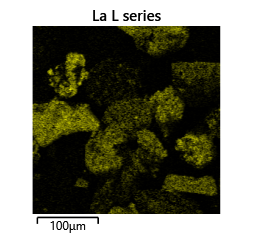


h

i


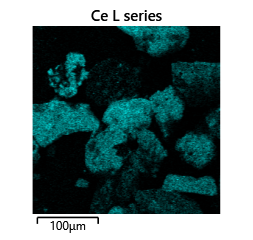

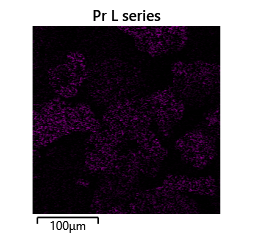


j

k


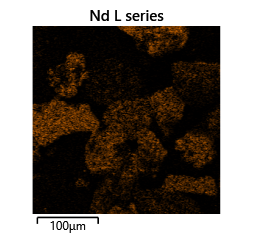

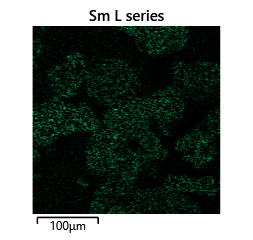


l

m

**Figure S-15.** SEM-EDS mapping of the mineral/chemical distribution and composition of high grade monazite sample. (a) The SEM overall image of the mapped area merged with the SEM-EDS elemental map. (b-m) The chemical distribution of each element in different mineral grains. The black regions in the SEM-EDS maps represent absence of an element of interest and the coloured area represent the areas on the surface where the related EDS signal was detected for that specific element.


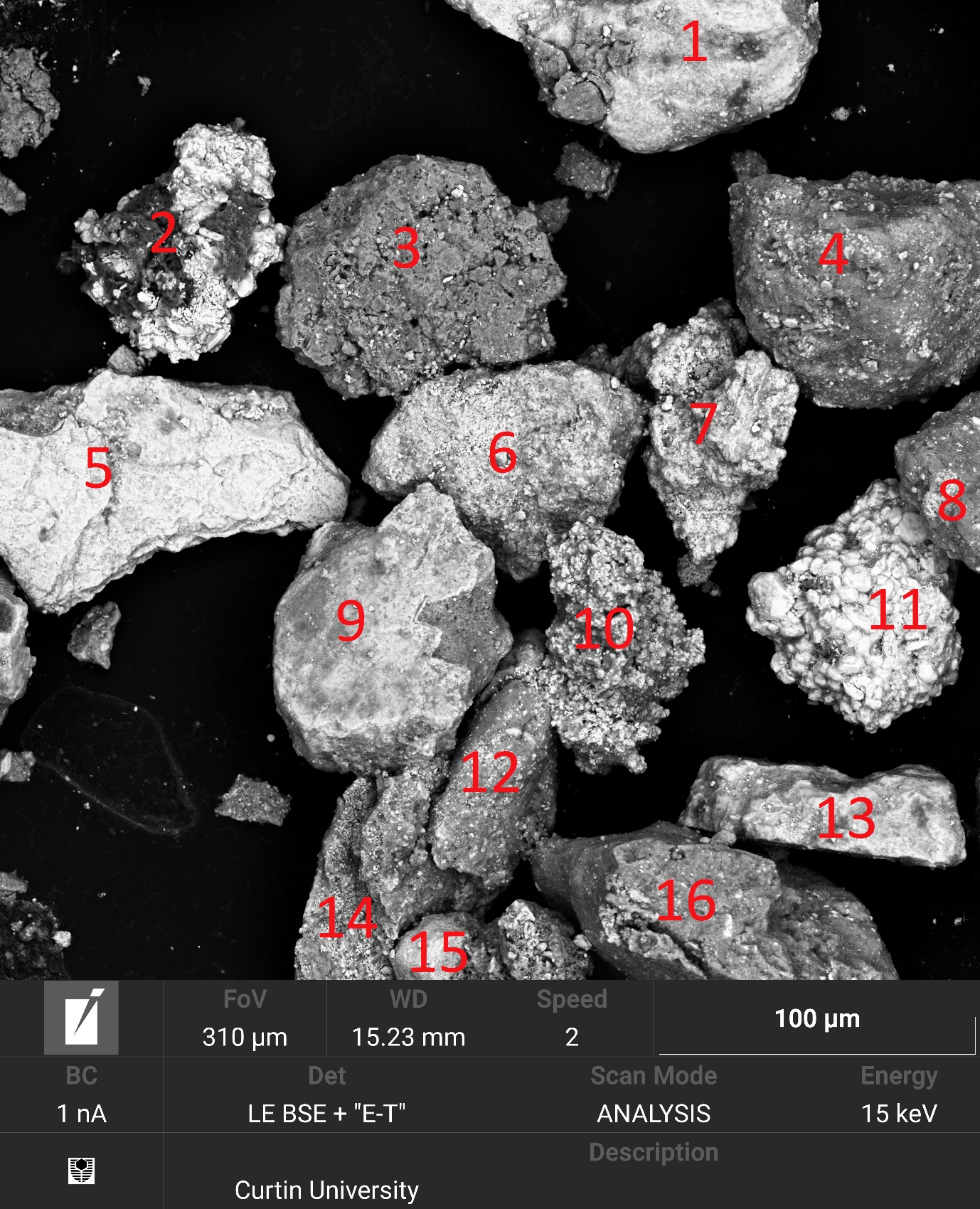


a

100 µm


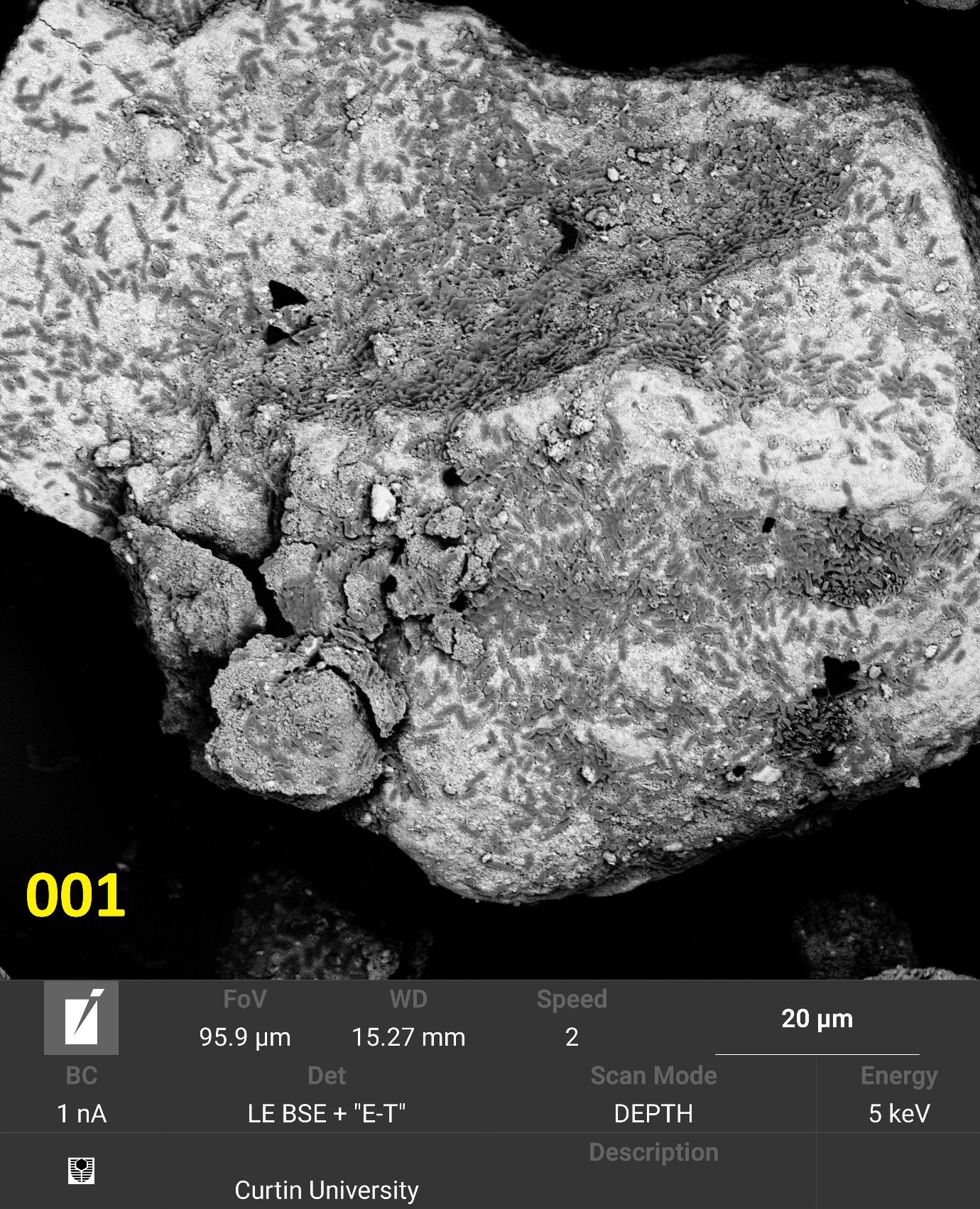


20 µm


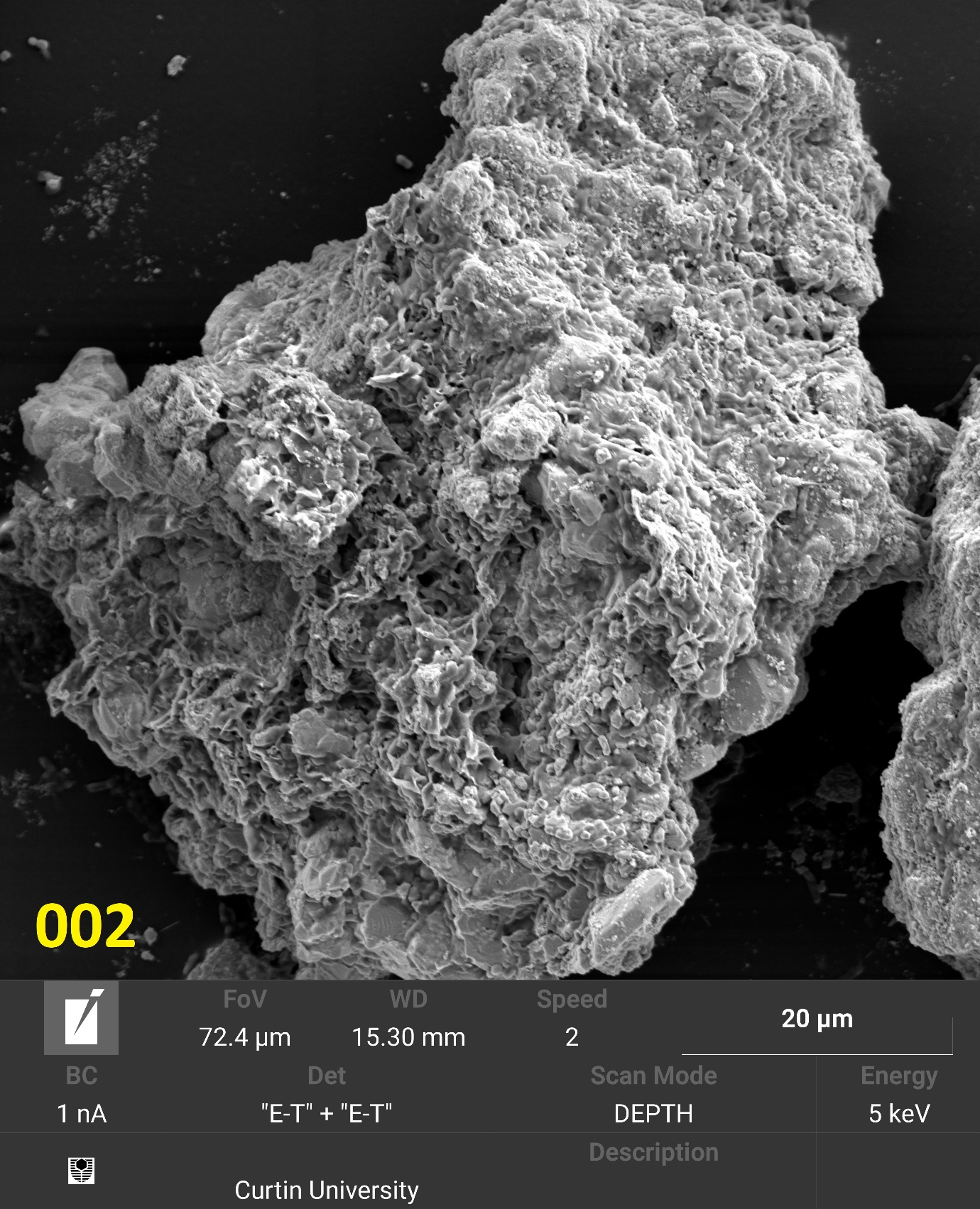


20 µm


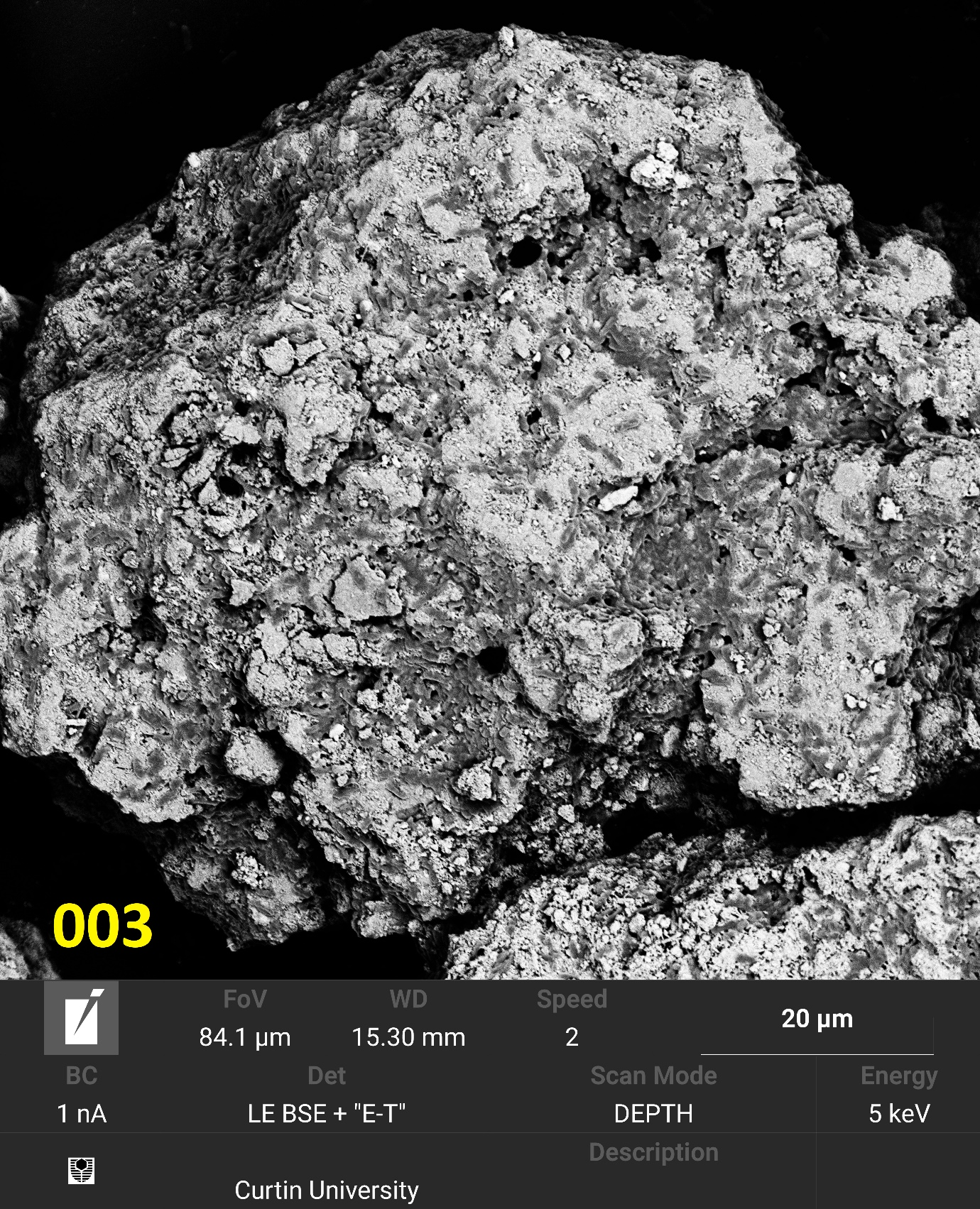


20 µm


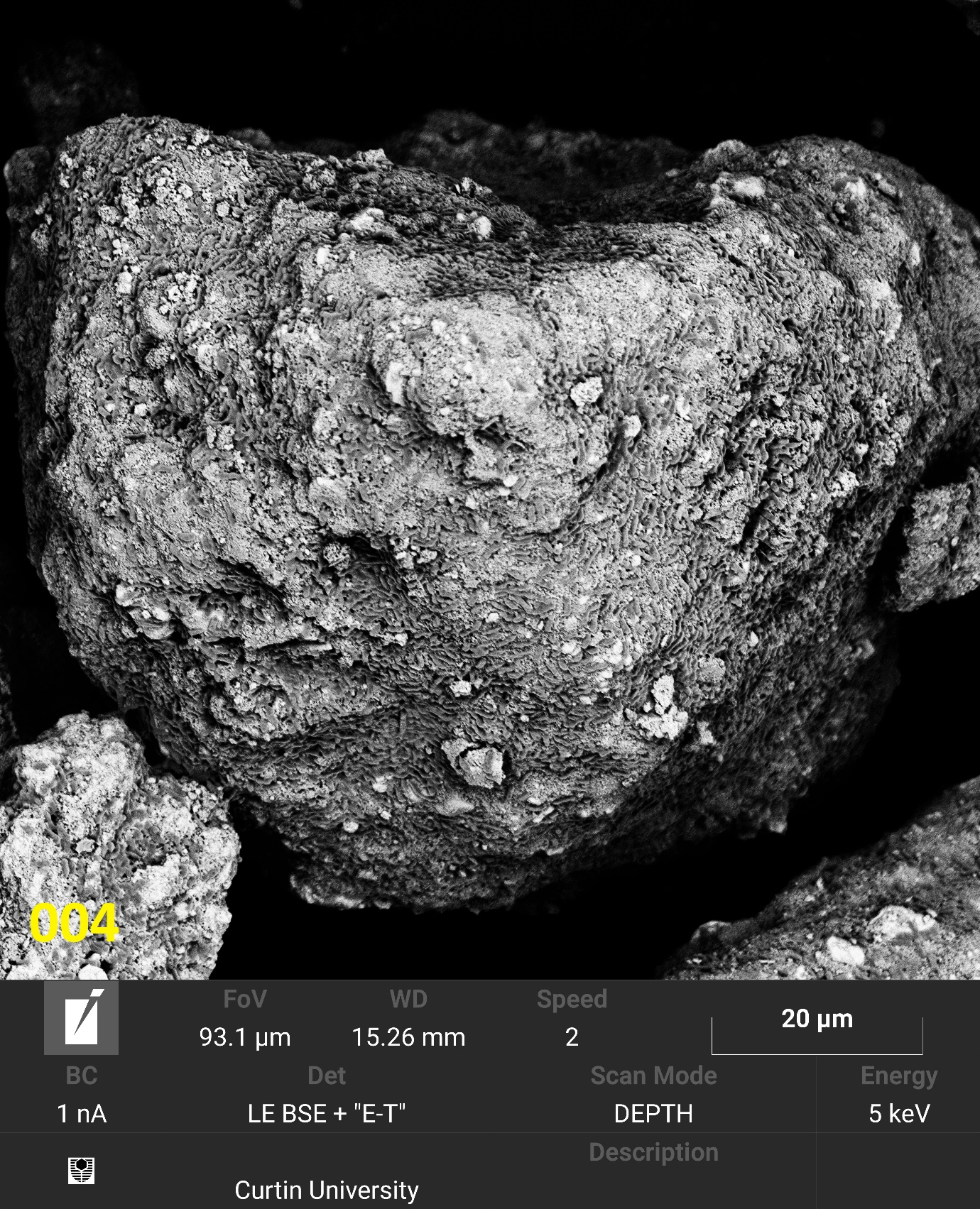


20 µm


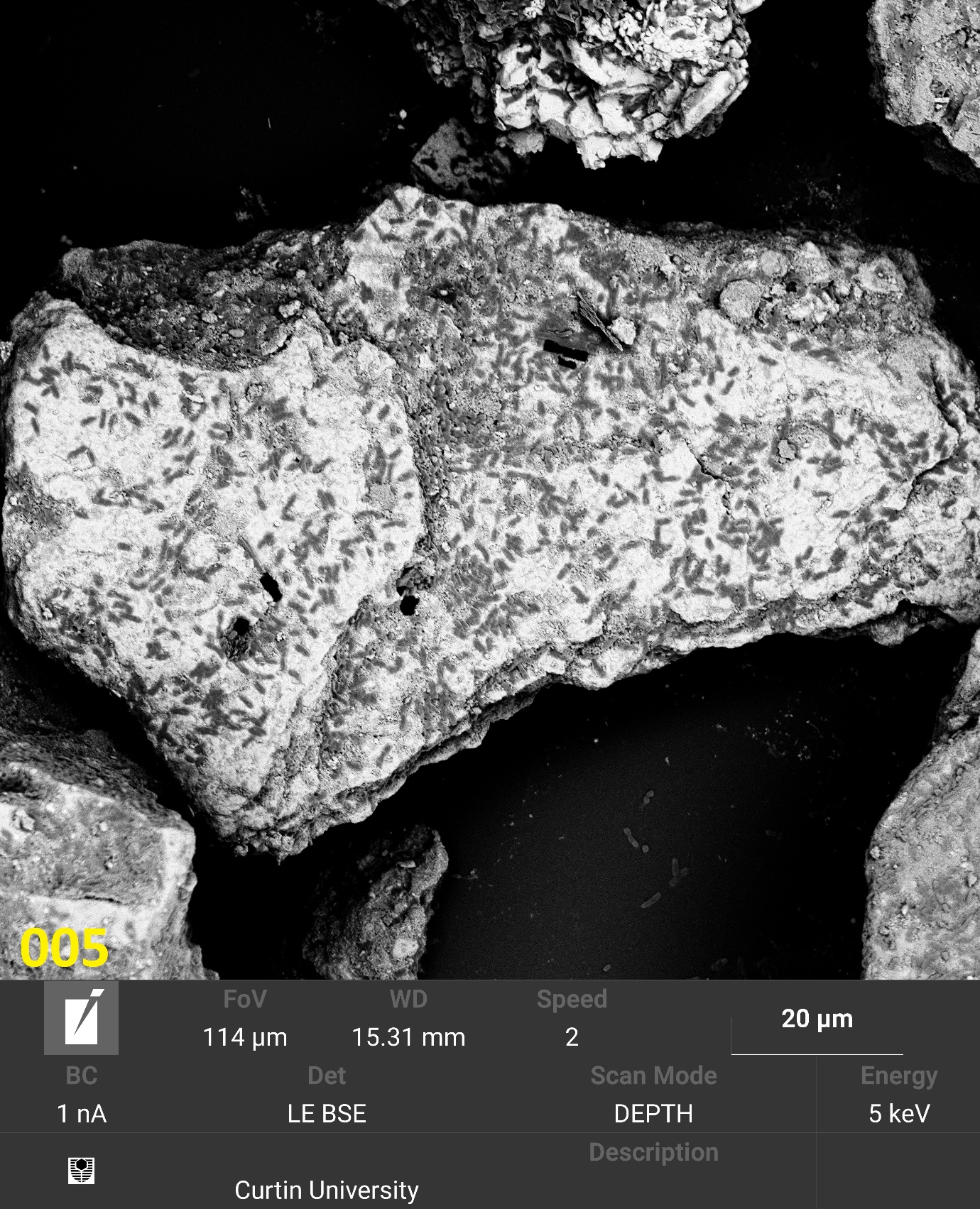


20 µm


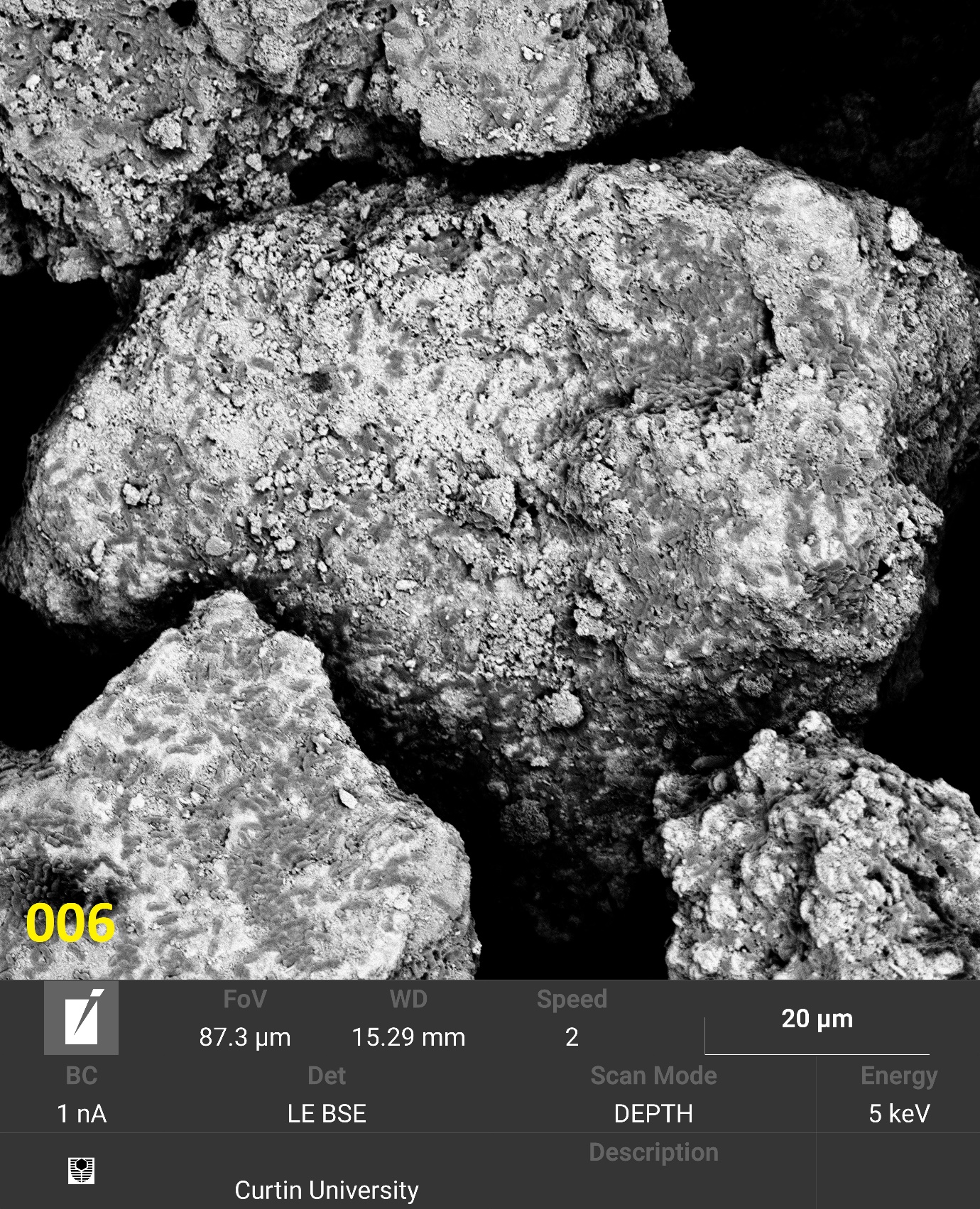


20 µm


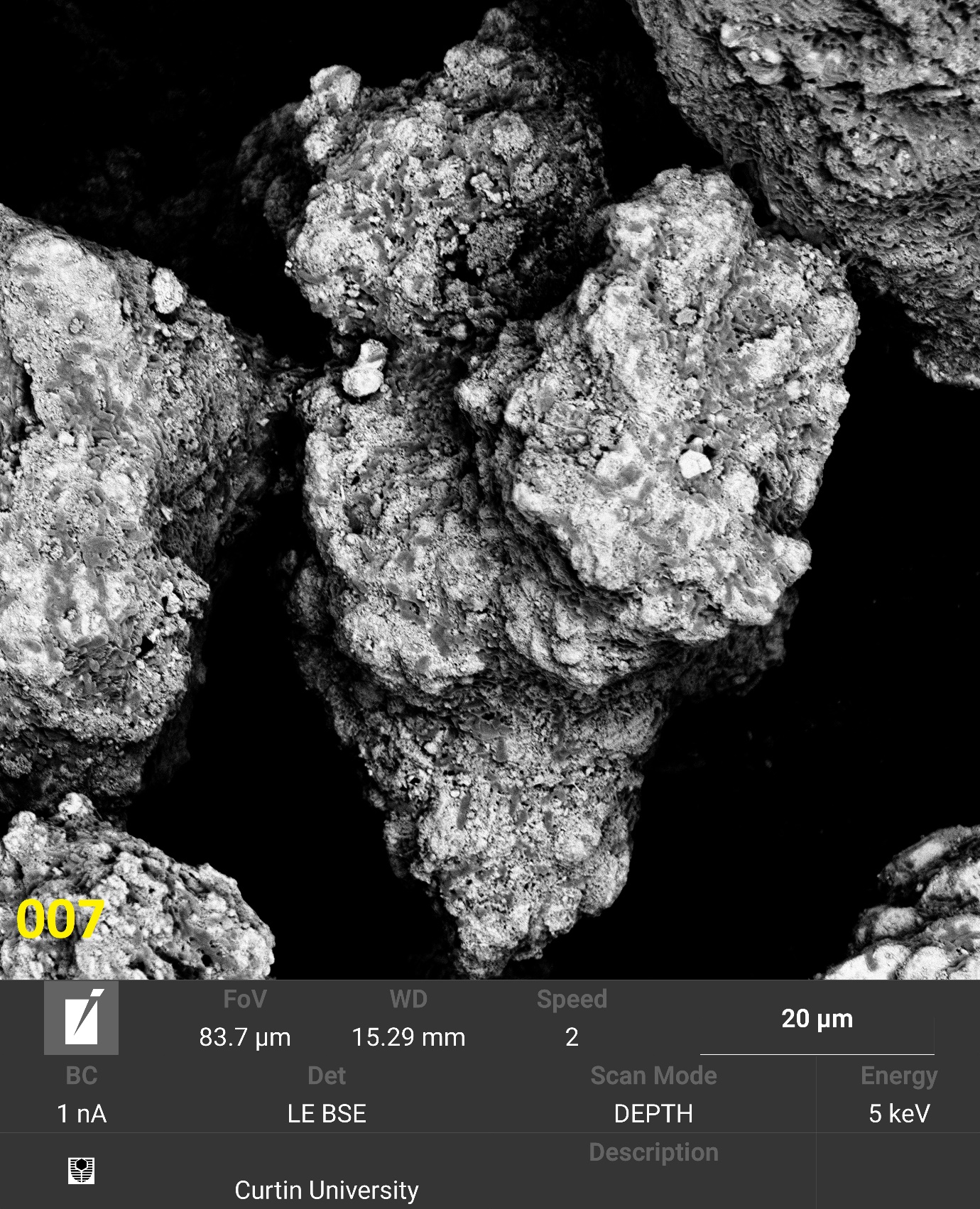


20 µm


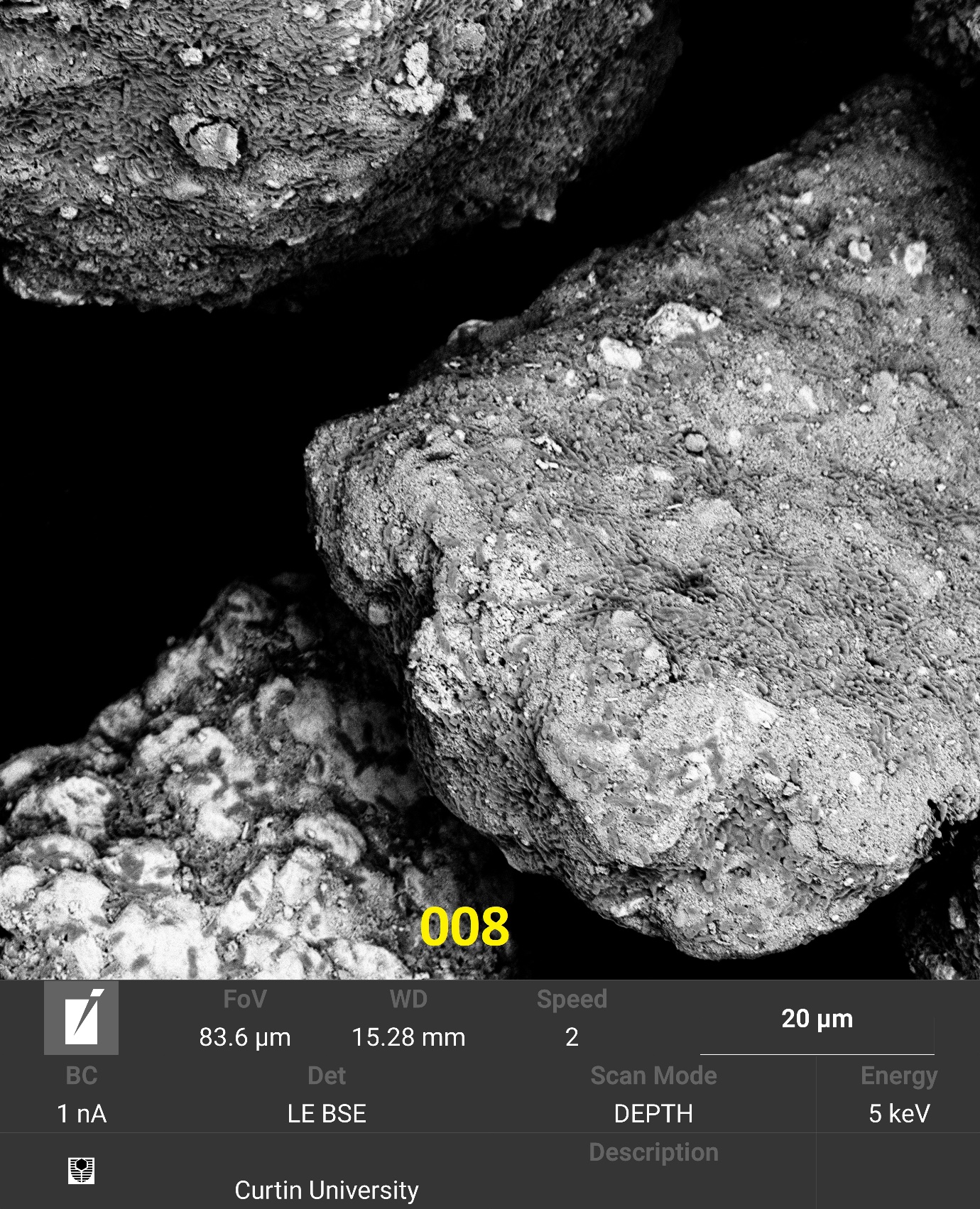


20 µm


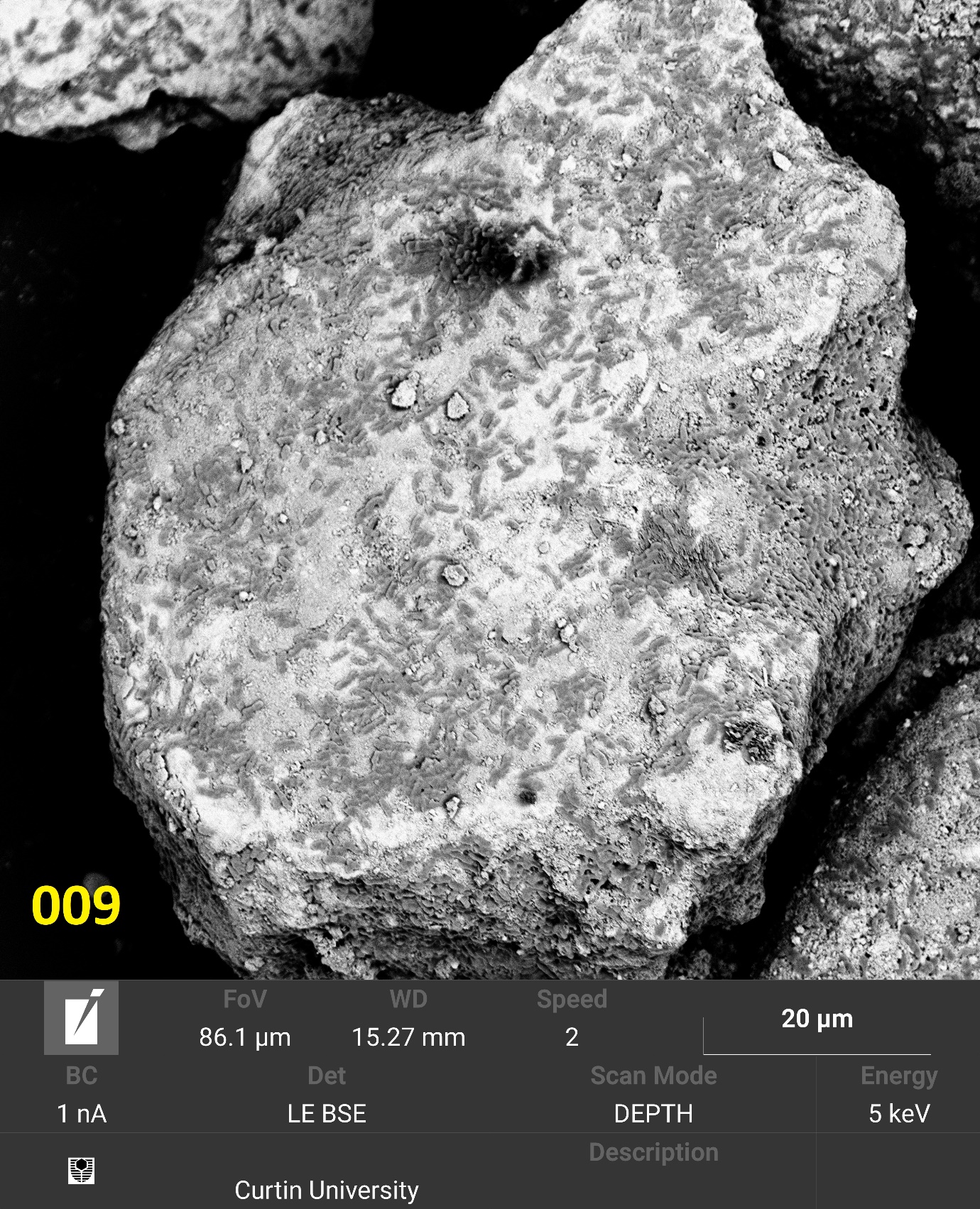

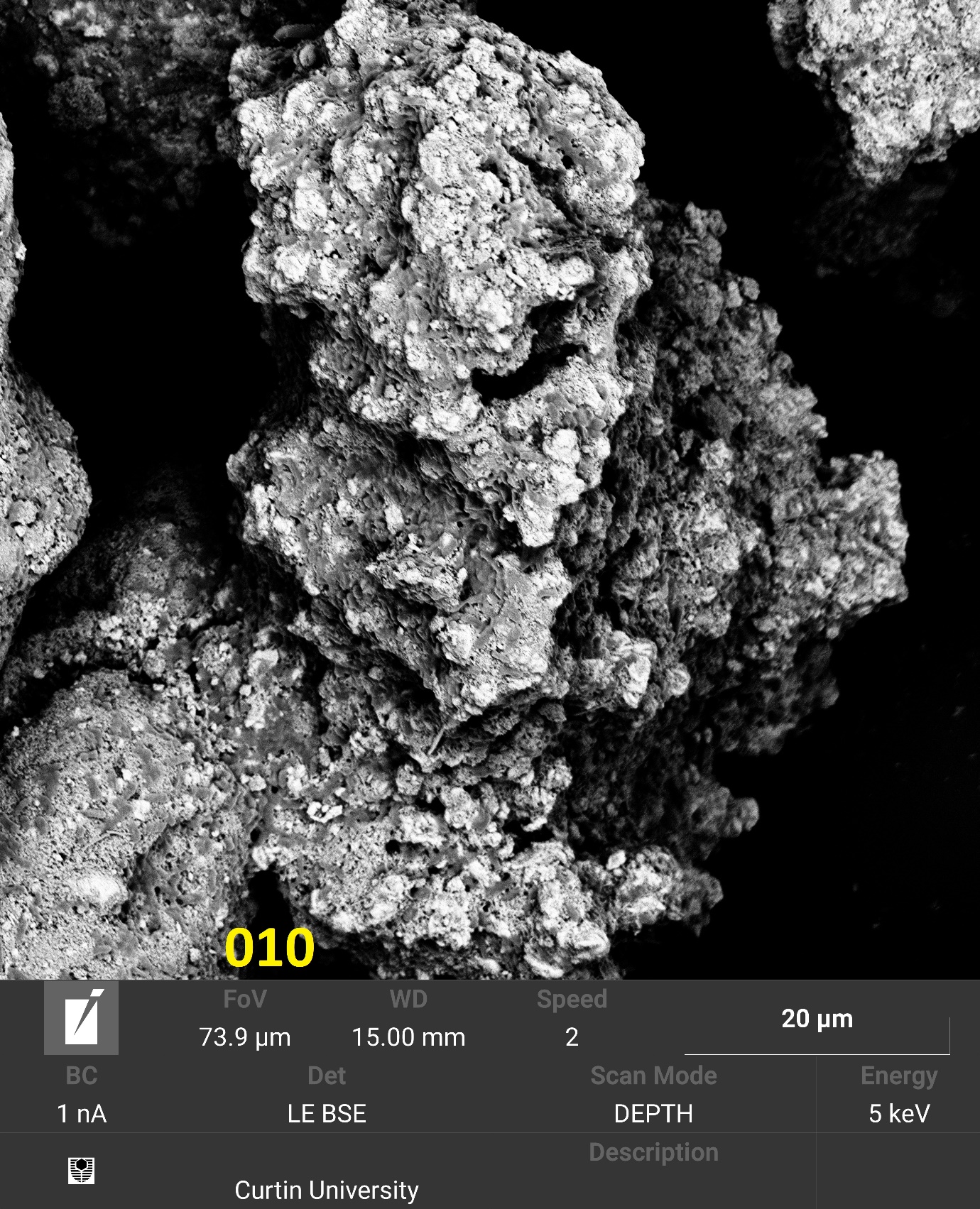
`
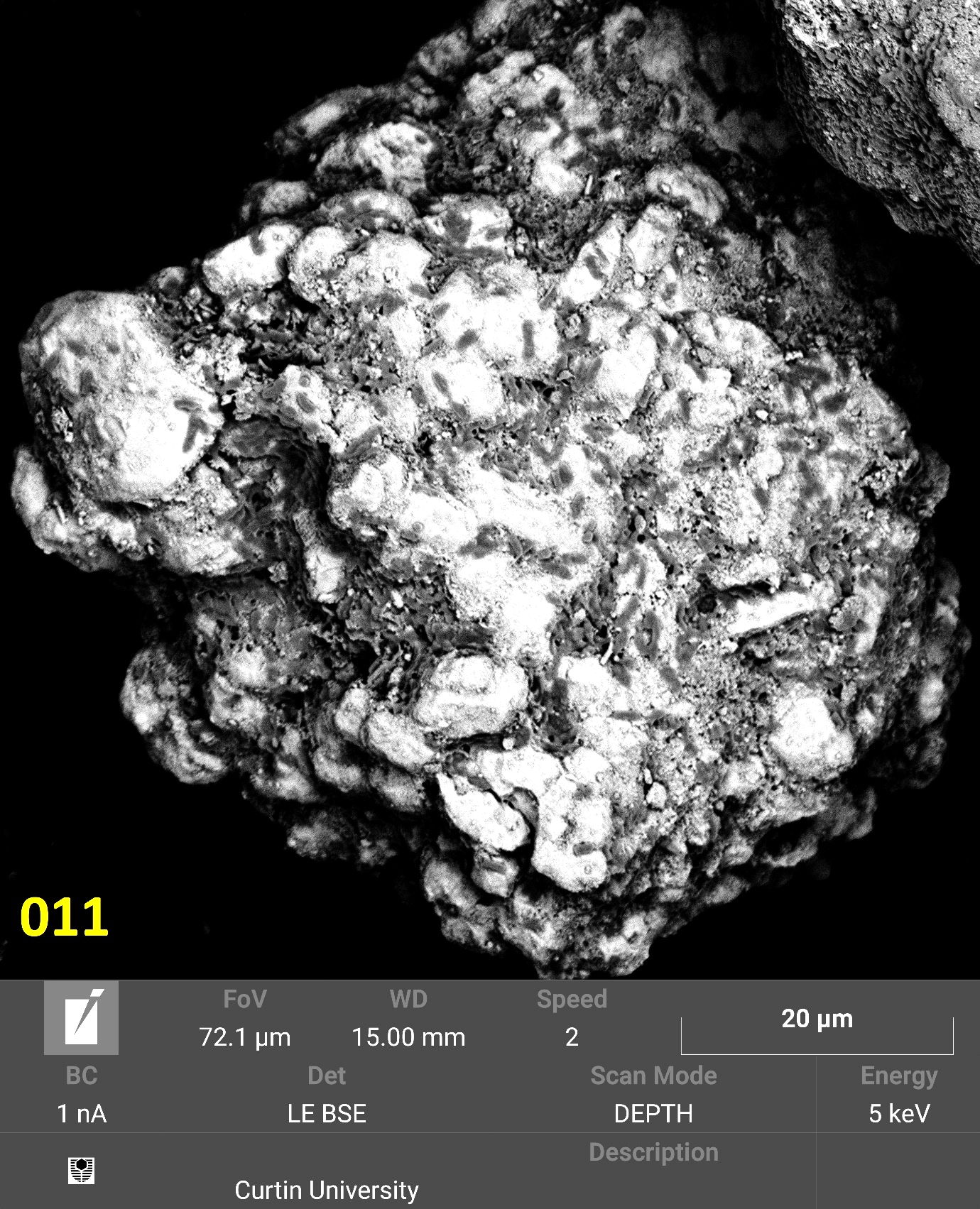

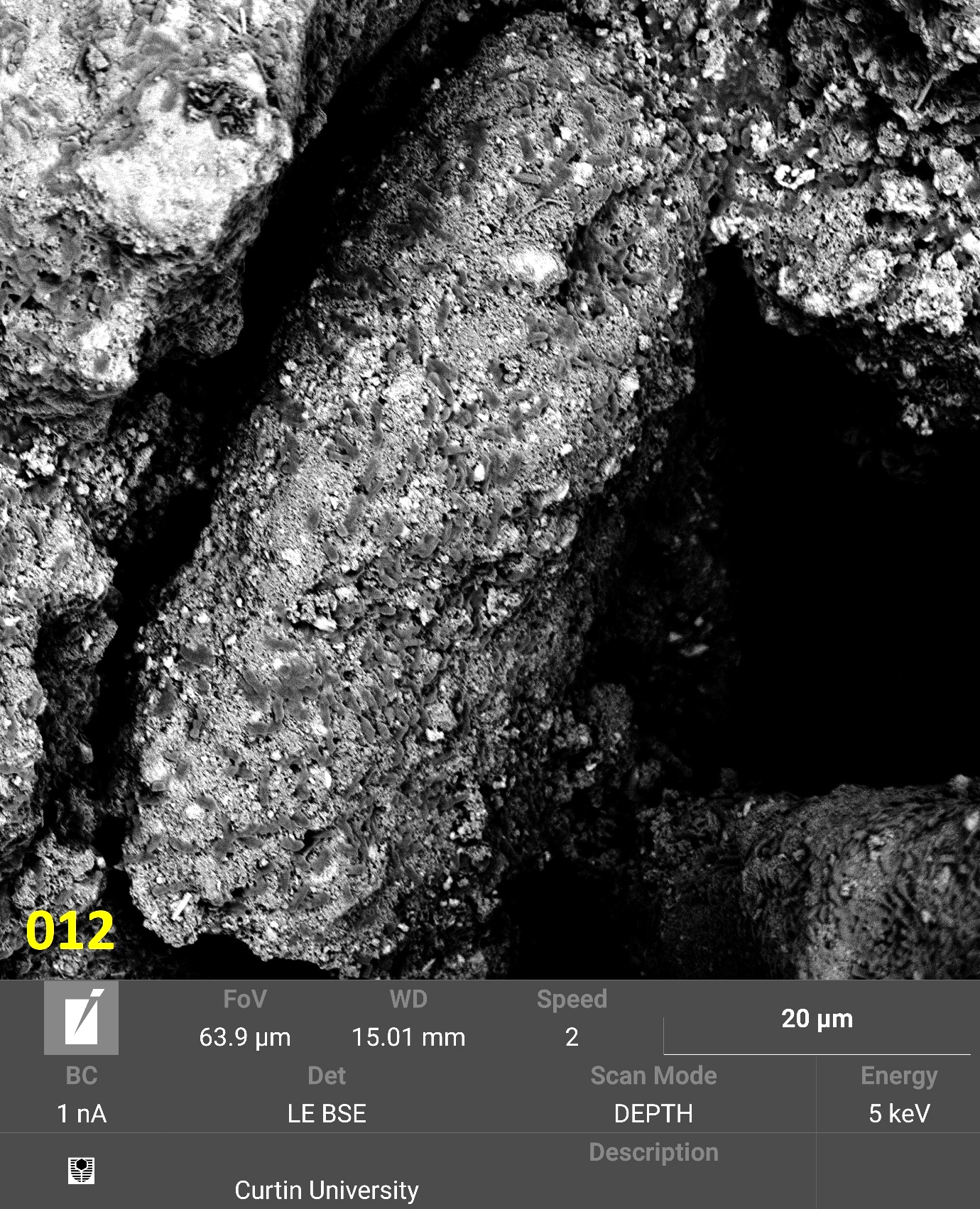

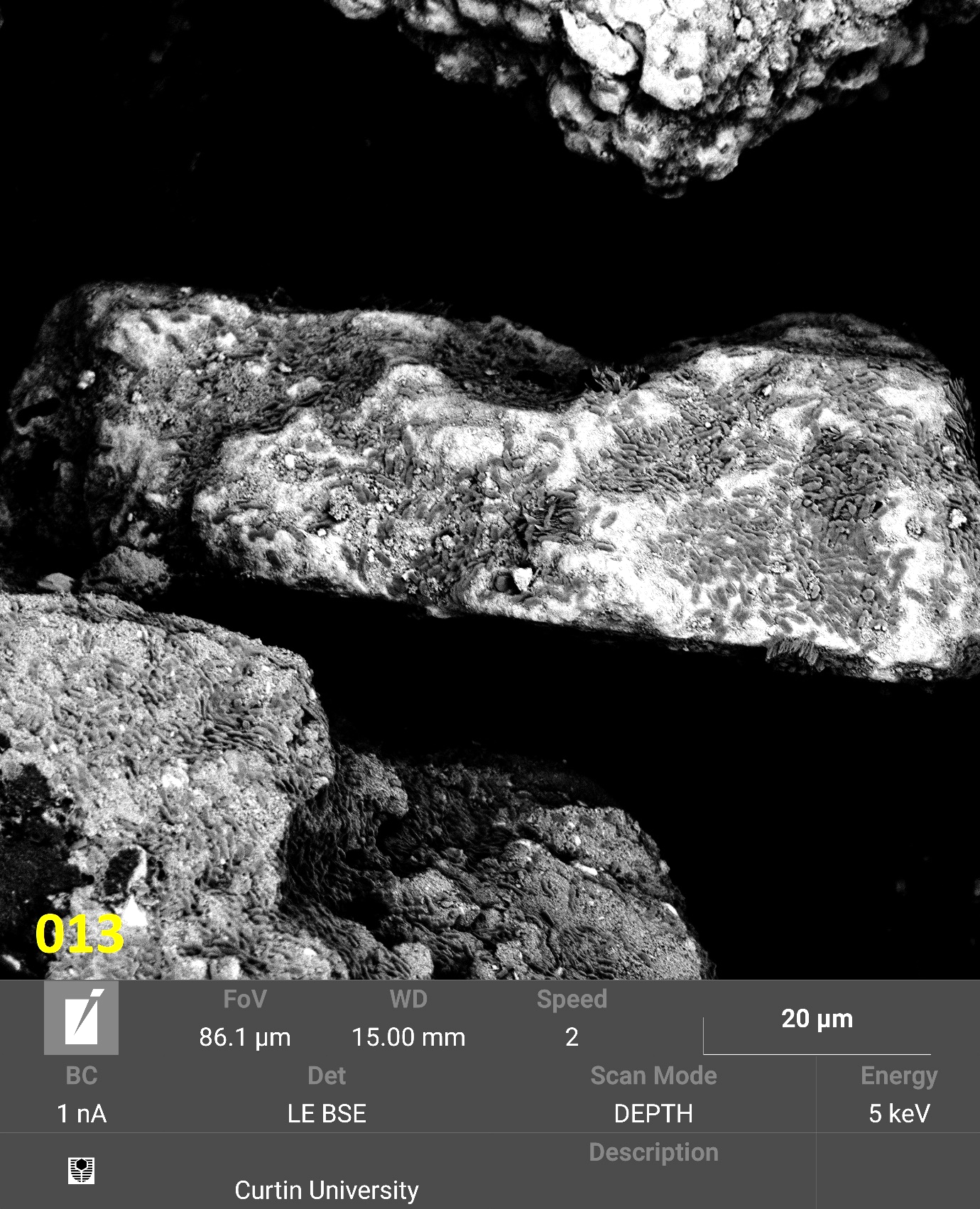

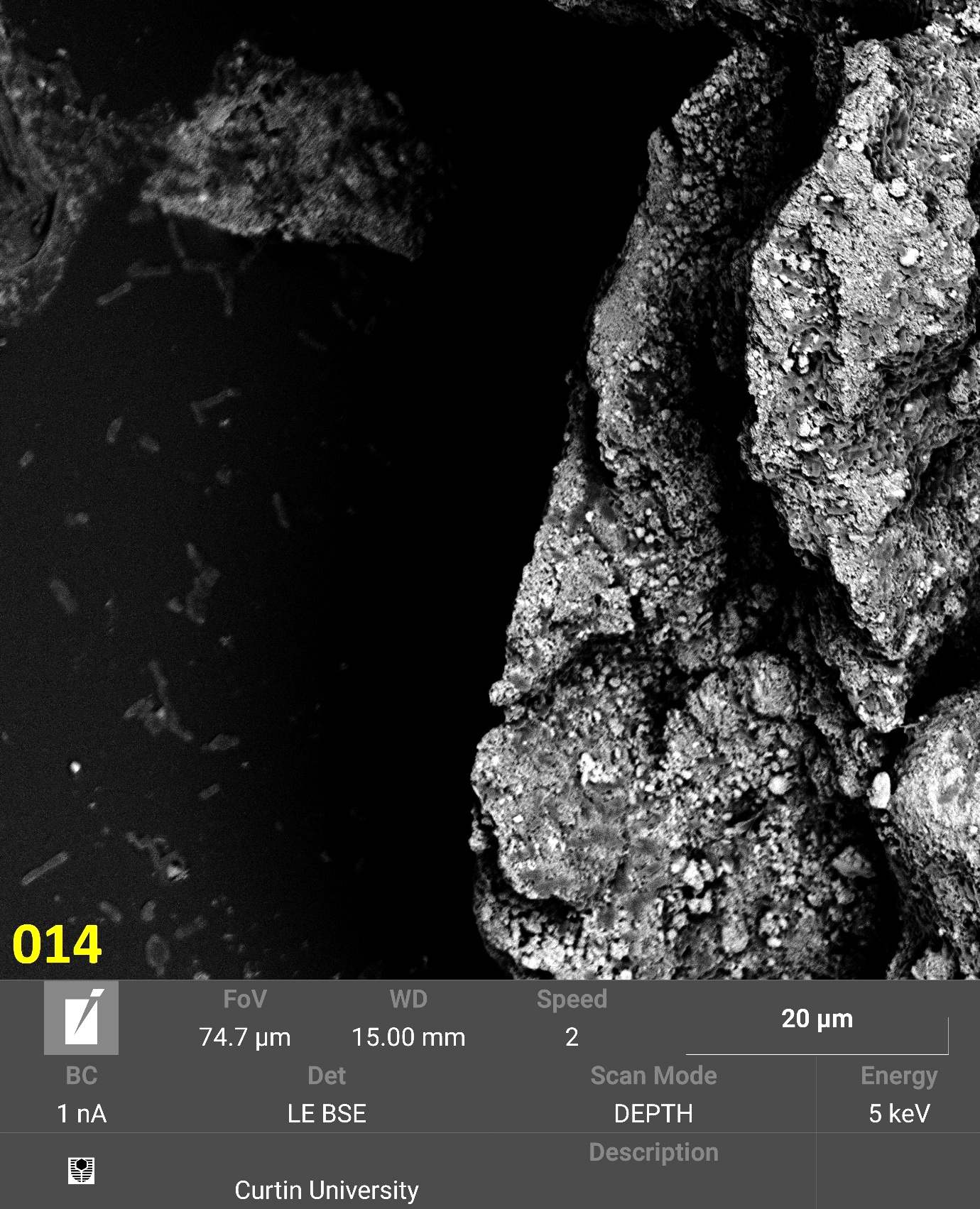

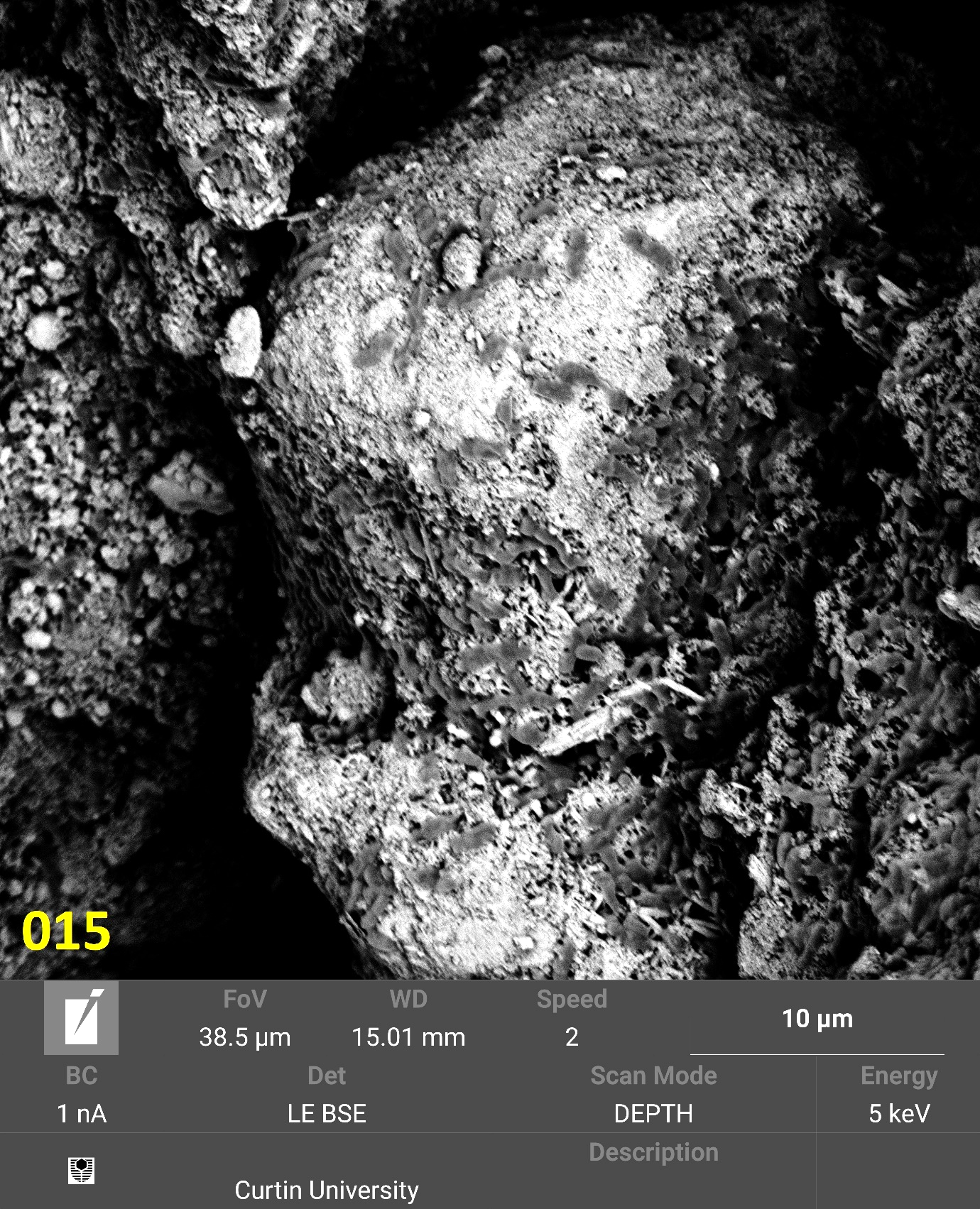

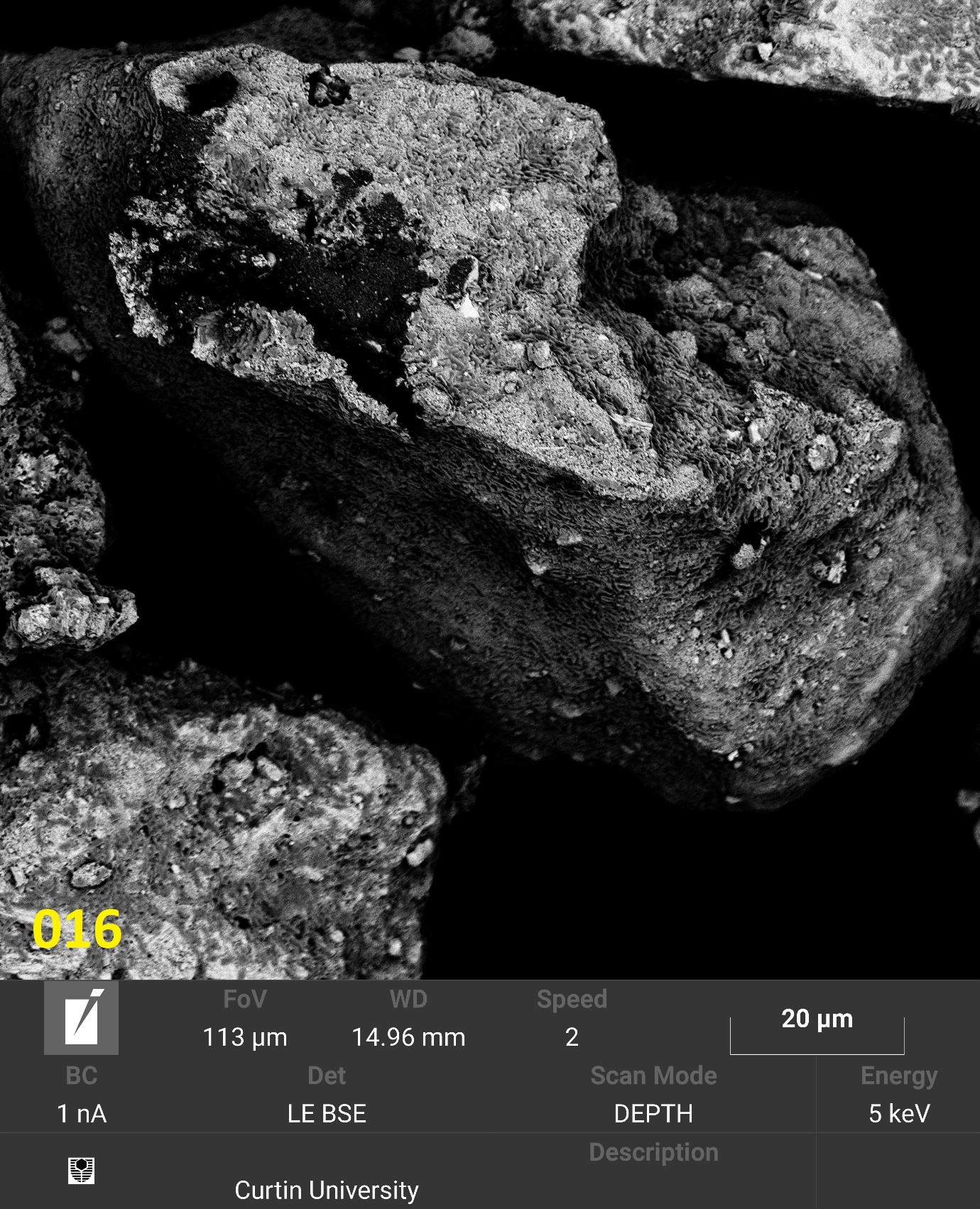


20 µm

20 µm

20 µm

20 µm

20 µm

20 µm

10 µm

20 µm

**Figure S-16.** Investigating *K. aerogenes* biofilm localization toward specific mineralogy or chemical composition. An overall backscattered electrons SEM image of the sample is provided (a) with each grain numbered for reference. The regions of the mineral surface with heavy element such as Ce or La are seen with brighter white-grey shades in the backscattered electrons SEM image and lighter element are seen darker. Another backscattered electrons SEM image of each grain was captured at higher magnifications and the referenced number of each grain (panel-a) is provided at the bottom of the image. *K. aerogenes* cells are the bacilli shaped grey features on the surface of the mineral grains.
